# Genome-wide CRISPR guide RNA design and specificity analysis with GuideScan2

**DOI:** 10.1186/s13059-025-03488-8

**Published:** 2025-02-26

**Authors:** Henri Schmidt, Minsi Zhang, Dimitar Chakarov, Vineet Bansal, Haralambos Mourelatos, Francisco J. Sánchez-Rivera, Scott W. Lowe, Andrea Ventura, Christina S. Leslie, Yuri Pritykin

**Affiliations:** 1 Department of Computer Science, Princeton University, Princeton, NJ USA; 2 Computational and Systems Biology Program, Memorial Sloan Kettering Cancer Center, New York, NY USA; 3 Cancer Biology and Genetics Program, Memorial Sloan Kettering Cancer Center, New York, NY USA; 4 Lewis-Sigler Institute for Integrative Genomics, Princeton University, Princeton, NJ USA; 5 Center for Statistics and Machine Learning, Princeton University, Princeton, NJ USA; 6 Weill Cornell/Rockefeller/Memorial Sloan Kettering Tri-Institutional MD-PhD Program, New York, NY USA; 7 Present address: David H. Koch Institute for Integrative Cancer Research and Department of Biology, Massachusetts Institute of Technology, Cambridge, MA USA

**Keywords:** CRISPR, Guide RNA, Off-targets, Burrows-Wheeler transform, Algorithm, Software, Web interface, GuideScan2

## Abstract

We present GuideScan2 for memory-efficient, parallelizable construction of high-specificity CRISPR guide RNA (gRNA) databases and user-friendly design and analysis of individual gRNAs and gRNA libraries for targeting coding and non-coding regions in custom genomes. GuideScan2 analysis identifies widespread confounding effects of low-specificity gRNAs in published CRISPR screens and enables construction of a gRNA library that reduces off-target effects in a gene essentiality screen. GuideScan2 also enables the design and experimental validation of allele-specific gRNAs in a hybrid mouse genome. GuideScan2 will facilitate CRISPR experiments across a wide range of applications.

**Supplementary Information:**

The online version contains supplementary material available at 10.1186/s13059-025-03488-8.

## Background

CRISPR-based technologies have transformed life sciences and shown promise in the development of therapeutics [1–3], and genome-wide CRISPR screens are routinely used for the unbiased identification of regulators of a diverse range of cellular phenotypes. However, the design of efficient and specific guide RNAs (gRNAs) for CRISPR-based genomic perturbations presents computational challenges. Unwanted gRNA off-targets can cause inefficient targeting as well as genotoxicity, and incomplete information about off-targets can result in misinterpretation of experimental results [4]. We previously developed GuideScan [5] for scalable gRNA design. GuideScan was extensively used by us and others for design of various CRISPR experiments [4–11]. We and others have demonstrated that GuideScan is more accurate than other tools in enumerating potential off-targets and estimating gRNA specificity, accounting for which improves the design and analysis of CRISPR experiments [4–7, 12]. It was observed that short-read alignment algorithms used by other gRNA design tools, while highly efficient for typical read count quantification tasks, do not exhaustively count suboptimal alignments or even multiple perfect alignments (without mismatches) [5, 13]. GuideScan overcame this limitation by using a custom retrieval tree (trie) data structure to preprocess and analyze the targetable genome. The trie is constructed and stored in memory and then used to enumerate all off-targets of all potential gRNAs, thereby assessing their specificity. The database is then constructed from all uniquely targeting gRNAs. Despite addressing the main limitation of previous gRNA design tools, GuideScan requires that the targetable space is pre-specified: for example, for CRISPR-Cas9, only 20-nucleotide long sequences (20-mers) followed by the protospacer-adjacent motif (PAM) sequence NGG are considered as primary targets, and those followed by NGG or a secondary PAM sequence NAG could be considered as off-targets. While the resulting gRNA database is stored on disk and allows for efficient access by genomic coordinates, the intermediate GuideScan trie data structure requires a large amount of memory for mammalian genomes (e.g., > 190 Gb for human genome assembly hg38), limiting the ability to manipulate the data structure or parallelize the gRNA database construction. For example, performing specificity analysis on gRNAs that are not in the GuideScan database would require loading this large trie data structure into memory, which is not feasible on most computers.

Here we present GuideScan2, a new gRNA design and analysis software that overcomes the limitations of GuideScan and significantly expands its functionality using a novel search algorithm. The new approach is based on the Burrows-Wheeler transform for indexing the genome, in combination with a new algorithm for simulated reverse-prefix trie traversals for searching gRNAs and their off-targets in the genome. GuideScan2 can be used for memory-efficient, parallelizable construction of high-specificity CRISPR guide RNA (gRNA) databases and user-friendly design and analysis of individual gRNAs and gRNA libraries for targeting coding and non-coding regions in custom genomes. GuideScan2 analysis identified widespread confounding effects of low-specificity gRNAs in published CRISPR screens and enabled construction of a gRNA library that reduced off-target effects in a gene essentiality screen. GuideScan2 also enabled the design and experimental validation of allele-specific gRNAs in a hybrid mouse genome. Thus GuideScan2 can be used for CRISPR experimental design and analysis across a broad range of applications.

## Results

**Fig. 1:**
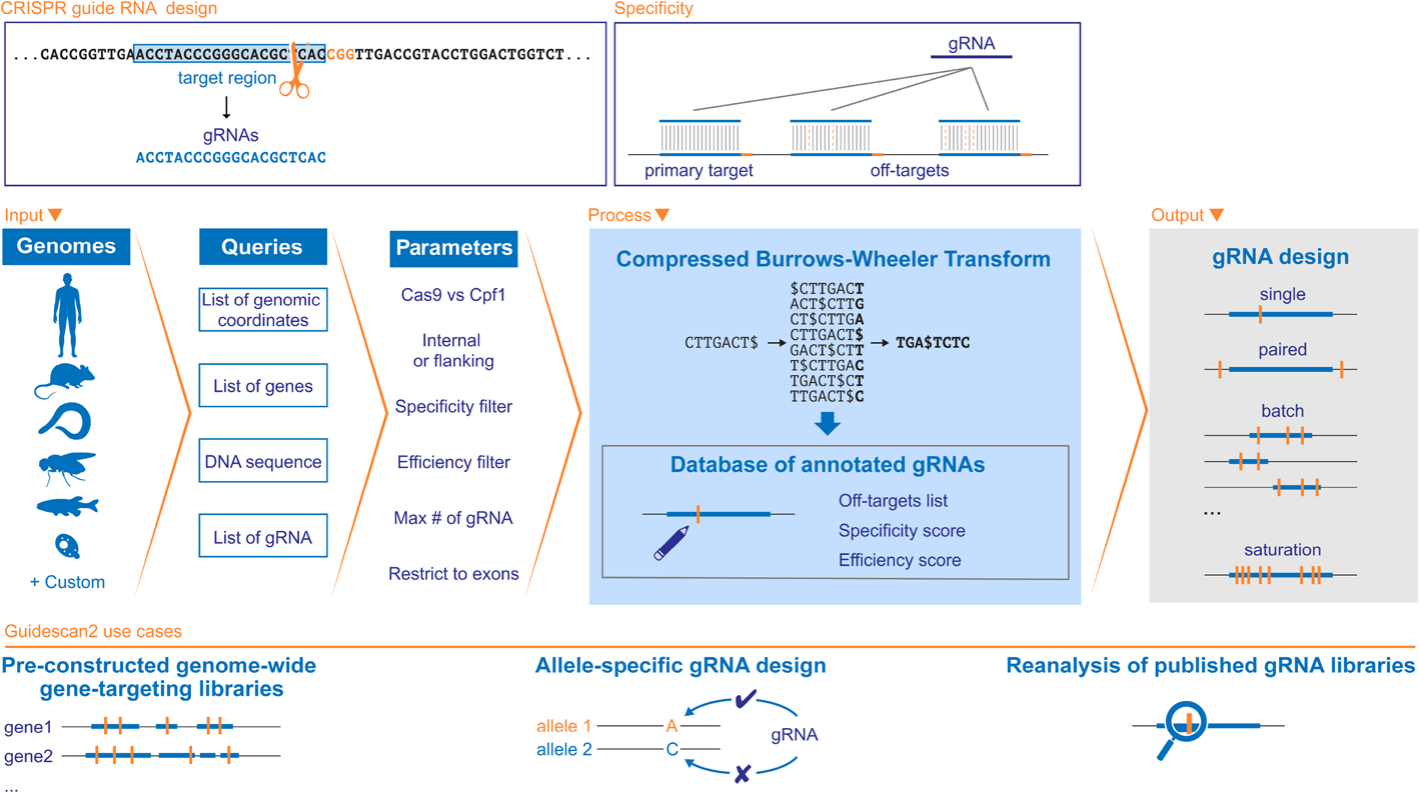
Overview of GuideScan2. GuideScan2 enables the design of gRNAs or gRNA libraries while exactly accounting for off-target effects. GuideScan2 CRISPR-Cas9 and CRISPR-Cas12a libraries are precomputed for standard genomes; databases for custom genomes can be constructed with the command line tool. GuideScan2 builds a memory-efficient compressed index based on Burrows-Wheeler transform to annotate all sufficiently specific gRNAs, enabling multiple design and analysis tasks

We present GuideScan2, a new gRNA design and analysis software that overcomes the limitations of GuideScan and significantly expands its functionality using a novel search algorithm. GuideScan2 uses simulated reverse-prefix trie traversals to enumerate off-targets. This is implemented by preprocessing the entire input genome into a lightweight index based on a compressed Burrows-Wheeler Transform (Fig. 1, “Methods” section, Additional file 1). Therefore, GuideScan2 is built upon the same efficient data structure as short-read aligners but is engineered to exactly account for off-targets. This approach does not require prespecifying the targeting rules, and the same index can be used for analysis and database construction for different gRNA lengths, PAM sequences, and off-target definitions, including mismatches or RNA or DNA bulges in gRNA-to-DNA alignments. The GuideScan2 index is memory-efficient (e.g., 3.4 Gb for hg38, 50$\times$ improvement over GuideScan) and fast to construct (30 min on a standard laptop, 65$\times$ improvement over GuideScan), enabling efficient online access and scalable search by gRNA sequence. At the same time, the resulting gRNA database is as accurate as GuideScan in enumerating all potential gRNA off-targets and estimating specificity. GuideScan2 functionality for processing the genome, for index and database construction and manipulation, as well as for gRNA design and analysis, is available through an open source command-line software package at https://github.com/pritykinlab/guidescan-cli [14, 15]. We also provide a user-friendly web interface for gRNA design and analysis at https://guidescan.com.

**Fig. 2:**
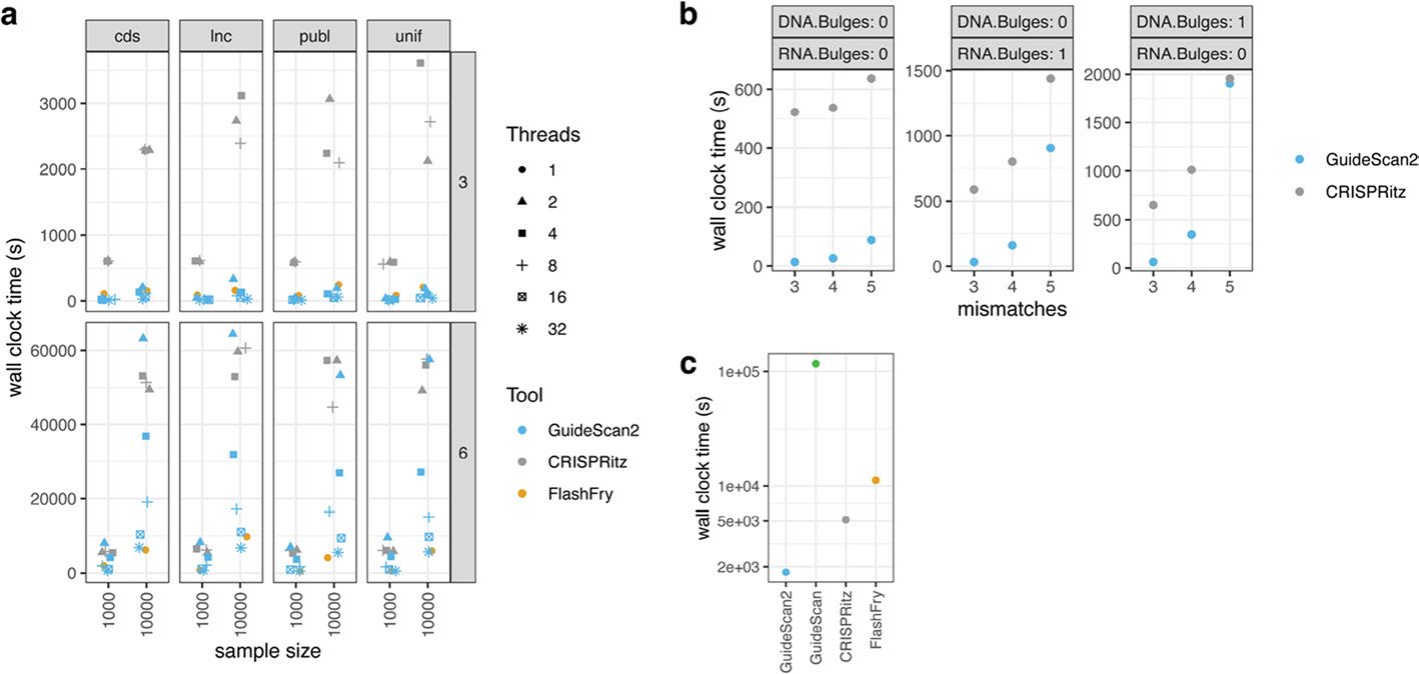
Comparison of GuideScan2 with other methods. **a** Run time comparison between different CRISPR gRNA design command line methods. GuideScan2 and other methods were used to find gRNA off-targets with up to 3 (top row) or up to 6 mismatches (bottom row). GuideScan2 and CRISPRitz were run with parallelization (number of threads specified by symbol shape). Inputs are random subsets of selected CDS-targeting regions (cds), long non-coding RNA genes (lnc), gRNAs from published libraries (publ), and gRNA sequences chosen uniformly at random (unif). **b** Run time comparison between GuideScan2 and CRISPRitz for gRNA off-target search allowing DNA or RNA bulges and variable number of mismatches, for 50 randomly selected gRNAs. **c** Comparison of genomic index generation time between the four methods. Indices constructed against hg38 reference for NRG PAM

Comprehensive qualitative and quantitative comparison demonstrated that GuideScan2 is more efficient, has more versatile features, and serves more use cases than other methods for CRISPR gRNA design and analysis (Fig. 2, Table S1, Methods section). In particular, GuideScan2 command-line tool is more efficient than other tools in indexing and searching genomes, and GuideScan2 web interface is the only tool allowing efficient genome-wide batch gRNA search queries by both sequence and genomic coordinates, including in the non-coding genomes. GuideScan2 reports gRNA specificities in nearly the same way as GuideScan (but is slightly more conservative in accounting for off-targets with alternative PAMs, e.g., NAG for Cas9, see Methods section). This GuideScan functionality of gRNA specificity scoring was previously used in a number of studies to account for or filter out gRNAs with low specificity [4–10, 12], and more recently GuideScan2 was used for the design of highly specific gRNAs for non-coding regulatory genome by the ENCODE consortium [16]. We observed that GuideScan2 specificities are significantly correlated with specificities measured with direct sequencing-based experimental methods (Spearman correlation 0.44 ($p < 0.001$), Additional file 1: Fig. S2, Methods section) [17].

**Fig. 3:**
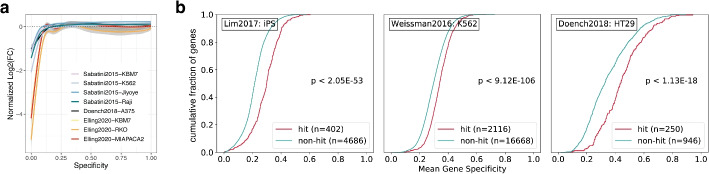
GuideScan2 reveals widespread confounding effects of low-specificity gRNAs in published CRISPR screens. **a** Reanalysis of published essentiality CRISPRko screens for non-essential genes (smoothed conditional means, “Methods” section). **b** Reanalysis of published CRISPRi screens. Comparison of gene-level specificity (mean over gRNAs per gene) for genes identified by the authors as hits and non-hits. Empirical cumulative distribution function; Kolmogorov–Smirnov test, two-sided

**Fig. 4:**
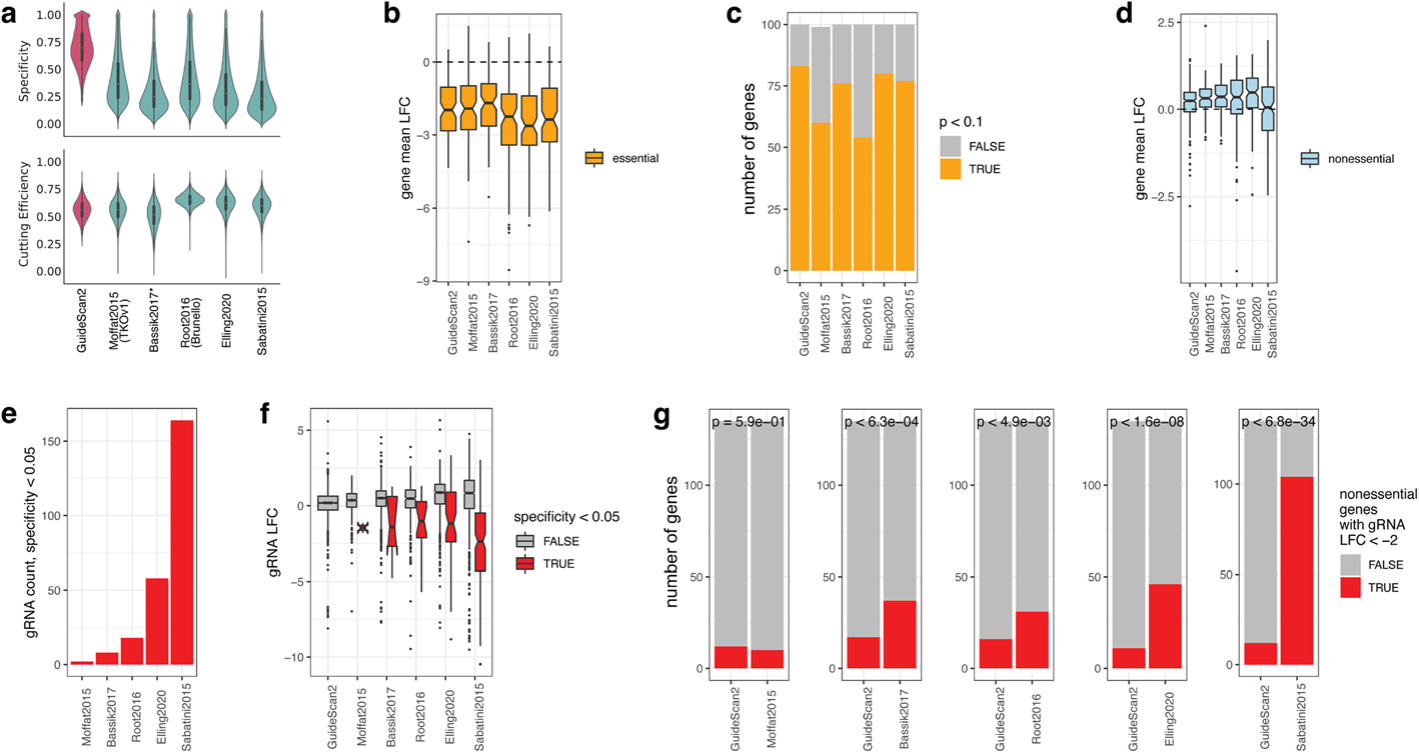
GuideScan2 enables design of new high-specificity gene-targeting gRNA libraries. **a** Comparison of new GuideScan2 human gRNA library against other libraries by specificity and cutting efficiency (mean over gRNAs per gene). Violin plot, boxplots (center line, median; box limits, upper and lower quartiles; whiskers, 1.5 $\times$ interquartile range). **b–g** Experimental results for the essentiality screen comparing new GuideScan2 gRNA library with other libraries. **b**, **d** Targeting effect (mean over gRNAs per gene) for essential and non-essential genes. **c** Number of essential genes identified as essential by gRNAs from each library (at $p < 0.1$, one-sided Wilcoxon test, “Methods” section). **e** Number of gRNAs with specificity below 0.05 targeting non-essential genes in each library. By design GuideScan2 does not have gRNAs with specificity below 0.05. **f** Targeting effect in non-essential genes for gRNAs depending on their specificity. **g** Number of non-essential genes in each pairwise library comparison that have at least one gRNA with strong negative effect (LFC $< -2$). Hypergeometric test.

To illustrate the value of GuideScan2, we set out to assess the performance of gRNA libraries used in published CRISPR gene essentiality screens (Fig. 3, 4a; Additional file 1: Figs. S3–S6). Thanks to its novel design, GuideScan2 allows straightforward enumeration of off-targets and evaluation of the specificity of all gRNAs used in each screen, including gRNAs that are not part of the GuideScan2 database. Consistent with previous observations by us and others [4, 5, 12, 18–21], we found that a substantial number of gRNAs in published CRISPR knockout (CRISPRko) screens [13, 18, 22–24] have many off-targets and consequently low specificity (Fig. 4a, Table S2; Additional file 1: Fig. S3). GuideRNAs with particularly low specificity can confound CRISPRko essentiality screens by producing strong negative cell fitness effects even for non-essential genes (Fig. 3a; Additional file 1: Fig. S3f) [15, 25], likely through toxicity due to a large number of non-specific cuts. We found a similar bias in some CRISPR inhibition (CRISPRi) gene essentiality screens, where gRNAs of lowest specificity targeting non-essential genes had significantly stronger cell fitness effect (which ideally is not expected for non-essential genes) than gRNAs with higher specificity (Additional File 1: Fig. S4a).

Importantly, we also identified a different previously unobserved confounding effect related to low gRNA specificity in genome-wide CRISPRi screens. Typically, such screens use multiple gRNAs for each gene in order to increase statistical power and compensate for occasionally unreliable gRNAs. However, we observed that in published screens [26–30], genes identified by authors as the main hits tended to have significantly higher average gRNA specificity than non-hits (Fig. 3b; Additional file 1: Fig. S4, S5). Similar specificity differences were observed when screen hits were defined using MAGeCK [31] in a uniform manner across datasets (Additional file 1: Fig. S6). This suggests that genes targeted by gRNAs with lower average specificity are systematically less likely to be called as hits of the screens. In some cases, the predictive power of average gRNA specificity for calling genes as hits of the screen was comparable with that of the strongest biological factors identified by the authors (Additional file 1: Fig. S4f). This newly identified confounding effect could present a major challenge for interpretation of results of genome-wide CRISPRi screens. A possible explanation for this effect could be in reduced inhibition efficiency at the intended primary target due to the dCas9 (dead Cas9, the main CRISPRi component) being diluted across an excessively large set of off-targets, as observed recently [32, 33]. However, further mechanistic studies are needed. Similar effects were observed for a CRISPR activation (CRISPRa) screen (Additional file 1: Fig. S5b, S6a).

To overcome potential problems with gRNA off-targets and low specificity in genome-wide screens, we designed new ready-to-use genome-wide CRISPR gRNA libraries targeting protein-coding genes in the mouse and human genomes, including six gRNAs per gene and complemented with safe-harbor-targeting and non-targeting gRNAs as controls (“Methods” section, Table S3). GuideRNAs from our libraries have higher predicted specificity than other libraries while having similar cutting efficiency (Fig. 4a, Table S2; Additional file 1: Fig. S3a). We expect that adoption of our GuideScan2 gRNA library for genome-wide CRISPR screens will help avoid the confounding effects of low-specificity gRNAs described above. Importantly, the strategy we used for designing this library (“Methods” section) can be extended to design high-specificity gRNA libraries in various contexts across genomes, including for non-coding regions [4, 16].

For experimental validation and comparison of the GuideScan2 library with five other gRNA libraries [13, 18, 22–24], we performed a multi-arm essentiality screen in human A549 cells [34] (“Methods” section, Table S4). The log fold change (LFC) of sequenced read counts for each gRNA in this experiment measures the cell fitness effect from CRISPR editing with this gRNA, with large negative values indicating a more deleterious effect as expected for essential genes. While all gRNAs in this experiment were used concurrently in a single screen in order to avoid batch effects and redundancy, they were designed in several experimental arms with different interpretation goals (and therefore cannot and should not be analyzed all together). As a positive control, in the first arm of the experiment we selected a random set of 100 essential genes and included all gRNAs for these genes from the six libraries into our screen. As expected, we observed that most of these gRNAs confer a strongly negative cell fitness effect that was comparable across libraries (Fig. 4b; Additional file 1: Fig. S7a–c), though the effect for GuideScan2 gRNAs was significant for larger number of essential genes than for gRNAs from other libraries (Fig. 4c). We also included negative control (non-targeting and safe-harbor-targeting) gRNAs in the experiment and confirmed that they do not result in lower cell fitness (Additional file 1: Fig. S7c, “Methods” section).

The main arm of the experiment was designed to assess the effect of using the low specificity gRNAs when targeting non-essential genes. This effectively consisted of five different subarms of the experiment, designed and aimed to be analyzed separately. For each of the five libraries that we compared against GuideScan2, we selected the 135 potentially most problematic genes, defined as non-essential genes for which gRNAs from that library had the lowest specificity. For this, genes were ranked by the average of the two lowest gRNA specificity values per gene. For the 135 genes selected for a library, we included into our screen all gRNAs for these genes from that library and from the GuideScan2 library, enabling direct pairwise comparison. We did this separately five times for each of the five libraries that were compared against GuideScan2. As expected for non-essential genes, these gRNAs overall tended to have little effect on cell fitness, consistently across libraries (Fig. 4d). However, we observed that a fraction of low-specificity gRNAs in each library conferred a strong deleterious effect on cell growth (Fig. 4e,f). We directly compared GuideScan2 to each of the other libraries, focusing on the number of non-essential genes for which at least one gRNA had a substantial deleterious effect (LFC $< -2$). For four of the libraries, this number was significantly higher than for GuideScan2, while for one library, Moffat2015 [22], the results were comparable (Fig. 4g). This is consistent with off-target analysis of the libraries performed using GuideScan2 where we found the Moffat2015 library to have the lowest number of off-targets (Additional file 1: Fig. S3b–d). Overall, this suggests that GuideScan2 is effective at enumerating off-targets and estimating gRNA specificity, and this information can be used to reduce the confounding effects of low-specificity gRNAs by designing libraries that avoid such gRNAs.

**Fig. 5:**
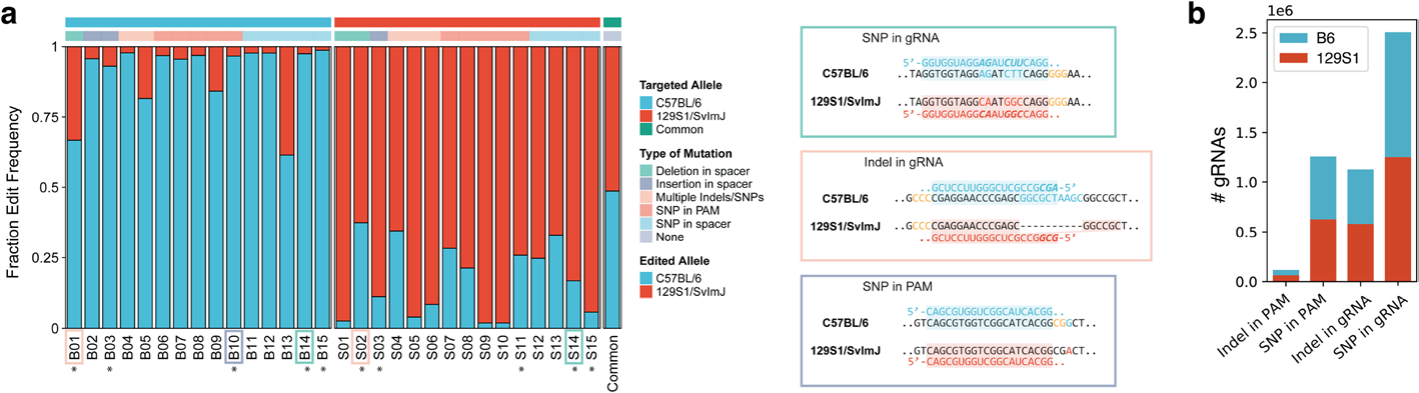
GuideScan2 enables design of a new gRNA library for allele-specific editing in a hybrid genome. **a** Experimental validation of selected C57BL/6- and 129S1/SvlmJ-specific gRNAs in mES cells of the F1 C57BL/6 $\times$ 129S1/SvlmJ hybrid mice. Asterisk marks manually chosen gRNAs, other gRNAs were chosen at random from high-confidence allele-specific gRNAs (“Methods ” section). **b** Total number of allele-specific gRNAs by category genome-wide (in millions). (B6, C57BL/6; 129S1, 129S1/SvlmJ)

To demonstrate GuideScan2’s flexibility in preprocessing and analyzing new genomes, we designed a genome-wide gRNA database for the F1 hybrid C57BL/6 $\times$ 129S1/SvlmJ mouse genome to enable allele-specific CRISPR targeting (Fig. 5, Table S5; Additional file 1: Fig. S8). The database includes gRNAs that are expected to target one of the two alleles more efficiently, due to SNPs or indels in the target protospacer sequence or PAM or due to larger structural rearrangements in one of the chromosomes, while controlling for all potential off-targets in all chromosomes of the hybrid diploid genome (Fig. 5). This database includes an approximately equal number of C57BL/6-specific and 129S1/SvlmJ-specific gRNAs, with an average of 92.6 C57BL/6-specific and 92.7 129S1/SvlmJ-specific gRNAs per 100Kb (Additional file 1: Fig. S8a). For 33% of protein-coding genes, we found at least one C57BL/6-specific and at least one 129S1/SvlmJ-specific exon-targeting gRNA (Additional file 1: Fig. S8b). When selecting a subset of allele-specific gRNAs using the more stringent requirement of SNPs or indels in the PAM rather than in the protospacer sequence, we still observed broad genome-wide coverage by such gRNAs (Fig. 5b). We experimentally validated 15 C57BL/6-specific and 15 129S1/SvlmJ-specific gRNAs, including some chosen at random across the genome from a pre-selected pool of higher-confidence gRNAs, and observed their high efficiency in allele-specific targeting (Fig. 5a, Table S6, “Methods” section). In sum, this database is a convenient resource for allele-specific genome editing in F1 hybrid C57BL/6 $\times$ 129S1/SvlmJ mice and demonstrates the use of GuideScan2 for gRNA design in custom settings.

To further illustrate GuideScan2’s efficiency in working with multiple genomes, we used it to build indices for several human genome references. After enumerating off-targets and estimating specificity for all gRNAs genome-wide, we found substantial specificity differences among the references for many gRNAs (Additional file 1: Fig. S9). This emphasizes the importance of choosing the most accurate reference genome for design and analysis of CRISPR perturbation experiments in specific biological systems, and GuideScan2 can serve as a valuable tool for such analysis.

## Discussion

We introduce a new algorithm and software GuideScan2 for the design and analysis of CRISPR gRNAs, gRNA libraries, and for gRNA database and genomic index construction in custom genomes. The GuideScan2 command-line software is based on a novel idea of directly using the Burrows-Wheeler transform for indexing the genome, which has not been previously used in gRNA design and analysis tools, in combination with a new algorithm for simulated reverse-prefix trie traversals. GuideScan2 has a new back-end and front-end command-line and web interface as compared with our previous method GuideScan. In benchmarking, we comprehensively demonstrate that GuideScan2 command-line and web interfaces are more efficient than other existing methods in indexing and searching the genome, and overall provide more versatile features and serve more use cases. Indeed, GuideScan2 can be used for efficient design and analysis of large screens for millions of genomic regions, without limitations on the position or size of such regions, or restrictions on the gRNA length, PAM sequence, and the number of mismatches or DNA and RNA bulges in off-targets.

In addition to introducing the GuideScan2 algorithm and software and benchmarking against other methods, we also present several GuideScan2 applications and novel findings obtained using GuideScan2, to further demonstrate the impact. This includes demonstration of previously unobserved confounding effects of gRNA off-targets in genome-wide CRISPRi screens; the design of a new GuideScan2 gene-targeting gRNA library, with experimental validation; the design of a new gRNA library for allele-specific targeting in hybrid genomes, with experimental validation; the demonstration of dependence of computationally predicted gRNA off-targets and specificity on a version of genome assembly. These results are interesting on their own, and would be computationally and analytically difficult or impossible to obtain using previous methods, including the previous version of GuideScan. We hope that the findings obtained using GuideScan2 will be followed up by future computational and experimental mechanistic studies by us and others to better understand and extend our observations, particularly regarding the confounding effects of gRNA off-targets.

## Conclusions

In conclusion, GuideScan2 is a new flexible and efficient CRISPR gRNA design and analysis tool with command line and web interfaces that will facilitate CRISPR experiments across a wide range of applications.

## Methods

### GuideScan2 algorithm

CRISPR-Cas systems can be used for targeted genome editing with RNA-guided endonucleases. They can be programmed to target a specific genomic location by designing a guide RNA (gRNA) with a particular short RNA sequence at its end, called the *spacer*. To elicit editing, the spacer sequence should be complementary or nearly complementary to the DNA sequence at the target location, called the *protospacer*, provided this sequence is adjacent to a particular protospacer adjacent motif (PAM). For example, CRISPR-Cas9 targets are generally thought to be determined by a 20-nucleotide spacer sequence at the end of the gRNA that is complementary to a DNA protospacer sequence followed immediately at the 3′ end by a PAM of the form NGG (resulting in more efficient targeting) or NAG (less efficient); here N stands for a “wildcard,” i.e., can match any nucleotide. Other natural and engineered CRISPR-Cas systems can vary in PAM sequence, PAM position with respect to the protospacer sequence, and requirements on the level of similarity between gRNA and the target. In what follows, unless otherwise noted, we will use CRISPR-Cas9 as our main example, but our approach is easily generalizable to other CRISPR systems. For simplicity we will associate the gRNA with its variable spacer sequence and will refer to it simply as the gRNA.

The task of gRNA design is, given a genomic region, to find gRNAs that can target anywhere in that region. Many potential gRNAs can target at multiple locations in the genome, though with varying efficiency. Typically a gRNA is designed to target a particular location with perfect complementarity. All other targets of this gRNA are then called *off-targets*. All information about off-targets of a gRNA is sometimes aggregated into a single measure of gRNA *specificity*, i.e., higher specificity means fewer off-targets. The main goal of gRNA design is typically to maximize gRNA efficiency at the primary on-target site while minimizing off-targeting. The major challenge is to balance between these two often conflicting goals.

Additional applications of gRNA design include: paired gRNA design to select two gRNAs targeting flanking sites of a genomic region of interest for cleavage of that region; saturation experiment design to exhaustively select all gRNAs to target anywhere within a selected extended genomic region of interest (e.g., a single gene); and batch library design to select the most effective gRNAs for each of a set of input regions (for example genes) for genomic screens. Another related task of gRNA analysis is, given a specific gRNA obtained from elsewhere (e.g., from a previously performed experiment), report information about its efficiency, specificity and potentially other properties for a particular genome. Other types of customized requirements for gRNA design and analysis are increasingly common and include: analyzing gRNAs of variable lengths; gRNAs with multiple perfect targets; gRNAs for new and custom genomes; gRNAs for allele-specific targeting.

To accomplish such tasks, our main approach is to preprocess the genome of interest to generate a genomic index. This genomic index can then be used for efficient queries reporting gRNA primary target and off-target information and for generation of the gRNA database for the given genome. The database contains gRNAs for the entire genome and information about off-targets of these gRNAs, according to a certain set of prespecified parameters, and provides the ability to efficiently search for gRNA information by genomic coordinates. The index can also be used to obtain additional information about gRNAs, e.g., about their more distant or non-conventional off-targets. It can also be used to search for information about any potential gRNAs, including those that were filtered out and excluded from the GuideScan2 database.

GuideScan used a retrieval tree (trie) data structure to preprocess the targetable space in the genome, i.e., all 20-mers followed by primary and secondary PAMs [5]. This trie was then used to construct a database, stored as a binary alignment map (BAM) file. This approach was guaranteed to identify all potential off-targets of a gRNA up to a certain number of mismatches. However, searching for non-conventional off-targets or for information about gRNAs that were not included in the database (e.g., those with multiple perfect occurrences (without mismatches) in the genome) was prohibitively difficult because such queries required manipulation of the trie, which was excessively large.

In contrast, GuideScan2 uses a data structure based on the Burrows-Wheeler transform (BWT) [35]. BWT compresses long sequences while allowing for efficient search of short subsequences [36]. The BWT index has been used for building software packages for short-read alignment such as Bowtie and BWA [37–39]. However, we previously showed that these packages cannot be used for efficiently enumerating gRNA off-targets [5], since their implementation is specific for their task and not easily adaptable to the genome-wide search required for our analysis. GuideScan2 relies on an efficient implementation of Wavelet Trees that utilize BWT based on rank-queries over the compressed sequence, and uses additional optimizations and heuristics [36, 39–43]. Complete technical details are provided in Additional file 1.

### Guide RNA scoring

As in GuideScan, we define the gRNA specificity score as an aggregate of the cutting frequency determination (CFD) scores [13] across all off-targets:

$$\begin{aligned} \text {Specificity}(g) = \frac{1}{1 + \sum \nolimits _{o \in \text {OffTargets}(g)}{\text {CFD}(o, g)}}. \end{aligned}$$

The specificity score takes a value between 0 and 1 (inclusive), and is closer to 0 for gRNAs with many and/or closely matching off-targets. As the CFD score is derived from empirical data generated only for length 20nt gRNAs [13], the specificity score is also defined only for length 20nt gRNAs.

Note that we decided to make GuideScan2 slightly more conservative than GuideScan in enumerating off-targets and computing the specificity score. GuideScan counts an off-target with an alternative PAM (e.g., NAG for Cas9) as having an extra mismatch, while GuideScan2 ignores mismatches in PAM for the purpose of off-target search. Therefore when searching for off-targets with up to a fixed number *k* of mismatches, GuideScan2 finds all off-targets that GuideScan finds, plus potentially additional ones (e.g., those with *k* mismatches in the spacer and a mismatch in PAM which GuideScan would count as $k + 1$ mismatches and will not return). This results in GuideScan2 off-targets for a gRNA being a superset of GuideScan off-targets, and therefore potentially GuideScan2 specificity score lower than GuideScan specificity score. However, for the vast majority of gRNAs, the difference in specificity scores between GuideScan and GuideScan2 is negligible. At the same time, GuideScan2-reported off-targets could be treated in customized manner in subsequent analysis if needed, including different treatment of mismatches in the spacer and in PAM.

### Comparison with experimental genome-wide estimates of specificities

Ideally for the goal of identifying gRNA off-targets and determining gRNA specificity, one would want to rely on direct experimental data for each gRNA of interest. However, this is practically infeasible. Existing experimental methods for direct genome-wide unbiased identification of gRNA off-targets, such as GUIDE-seq, VIVO, TTISS, CIRCLE-seq, Digenome-seq, DISCOVER-seq, ONE-seq, and others [44, 45, 17, 46–49], are not easily scalable and have been so far applied to a limited pool of gRNAs. Therefore GuideScan2 adopts a commonly used previously published formula for defining gRNA specificities [50] based on CFD scores [13].

A comprehensive comparison of gRNA specificities obtained from a variety of deep unbiased genome-wide profiling methods against the specificity estimates obtained using CFD scores and GuideScan2-identified off-targets is beyond the scope of this study. However, we performed a limited-scale comparison of gRNA specificity predictions obtained by GuideScan2 and CFD against experimentally derived gRNA off-targets and specificities using Tagmentation-based tag integration site sequencing (TTISS), a modified version of GUIDE-seq [17]. We obtained TTISS data for 59 gRNAs used with nine CRISPR enzymes, including wildtype Cas9 and eight Cas9 specificity variants [17]. Note that these 59 gRNAs were taken from the GeCKO library [51] and thus were already optimized to avoid off-targets. For each gRNA off-target, we calculated its relative frequency as the ratio of the TTISS read count for this off-target and the total read count across all off-targets for this gRNA when used with wildtype Cas9 (both read counts averaged over the replicates). Then these scores were used instead of CFD scores to define experimentally derived gRNA specificity. (Note that this is equivalent to calculating frequency of TTISS reads for the primary target of a gRNA among all TTISS reads for this gRNA.) Using GuideScan2 we also enumerated all potential off-targets for these gRNAs, and aggregated it into a specificity value using the above formula [50]. The default was to consider all off-targets with 3 or fewer mismatches between gRNA and protospacer. We observed a significant Spearman correlation of 0.44 between the experimentally defined and GuideScan2-estimated specificities across the 59 gRNAs. This was consistent with the similar comparison between TTISS and GuideScan specificities in the original TTISS paper where GuideScan showed higher correlation than another method providing gRNA specificity scores (GuideScan, *n* = 59, *R* = 0.408, CRISPR ML, *n* = 47, *R* = 0.111) [17]. We also calculated this correlation for other Cas9 variants, but the correlation for the wildtype Cas9 was the largest. Then we also calculated the correlation of TTISS-derived specifcities with the modified values of GuideScan2-estimated specificities taking into account all off-targets with up to *N* mismatches where *N* was 4, 5, or 6. The correlations for this modified GuideScan2-estimated specificities were lower than for the main definition of GuideScan2-estimated specificities with $N = 3$. GuideScan2 specificities for this analysis were calculated using the reference human genome (hg38, patch 13). These results are consistent with the previous comparison of GuideScan specificity values with GUIDE-seq results demonstrating high correlation [4].

### Comparison between GuideScan2 and other command-line tools for gRNA design and analysis

We compared the qualitative features of several command line CRISPR gRNA design and analysis tools including GuideScan2, GuideScan [5], CasOFFinder [52], FlashFry [53], CALITAS [54], CRISPRitz [55], and CRISPOR [56] and presented the results in Table S1.

Next, we compared the running time and memory usage of gRNA off-target enumeration between GuideScan2 and two other state-of-the-art methods, CRISPRitz and FlashFry. Note that the CRISPRitz publication [55] reported that both CRISPRitz and FlashFry were more efficient than CasOFFinder, making a comparison to CasOFFinder unnecessary. The other methods CALITAS and CRISPOR were not selected as they do not provide functionality to perform batch queries.

To measure the performance of off-target enumeration, we generated a set of 38,521 gRNAs to use in our timing comparisons that represent four distinct sets of gRNAs. The first set, consisting of 10,000 gRNAs and referred to as the (publ) published set, was drawn uniformly at random from the previously published CRISPRko, CRISPRi and CRISPRa libraries analyzed in this paper. The second set, consisting of 9385 gRNAs and referred to as (cds) CDS-targeting gRNAs, were also drawn uniformly at random from this same set of gRNAs, with the constraints that each gRNA must cut within a CDS (coding sequence) region and that no two gRNAs are in the same CDS region. The third set, consisting of 9136 gRNAs and referred to as the (lnc) long non-coding RNA targeting gRNAs, were drawn uniformly at random from the published gRNA library [26] with the constraint that no two gRNAs are in the same LNC region. The final set, consisting of 10,000 gRNAs and referred to as (unif) uniformly random gRNAs, were chosen uniformly at random from the set of all 20-nucleotide-long sequences.

Since GuideScan2, CRISPRitz, and FlashFry each construct a genomic index prior to search, a one time operation, we first measured the time to construct this index against the human reference genome hg38. Then, for each set of gRNAs, we measured the wall clock time of enumerating the off-targets within a mismatch neighborhood of *k* of all the gRNAs in the set across all tools. We performed this measurement across a range of mismatch parameters $k = 0, 1, 2, 3, 4, 5, 6$ and with varying the number of threads $t = 1, 2, 4, 8, 16, 32$. We used the PAM NRG (i.e., NGG or NAG) for all of these tests. Concurrently, we measured the maximum memory usage of the tools in each run.

For a smaller subset of 50 gRNAs, drawn uniformly at random from our published (publ) set, we measured the wall clock time of enumerating off-targets when also allowing RNA and DNA bulges, using GuideScan2 and CRISPRitz. We used a range of mismatch parameters $k = 3, 4, 5$ and three different settings of RNA and DNA bulge search parameters. All tests were performed on Princeton University’s *Ionic* research computing cluster, with 32 dedicated cores for each run.

We also compared the resulting off-targets and observed that they were identical or nearly identical between methods when used with matching parameters ensuring the same underlying definition of off-targets. For example, for a random set of 50 published (publ) gRNAs, we compared off-targets found by CRISPRitz and GuideScan2 with different sets of parameters. When searching for off-targets with up to 5 mismatches, 153,569 common off-targets were found by the two methods, 2 off-targets exclusive to GuideScan2 and none exclusive to CRISPRitz. When searching for off-targets with up to 5 mismatches and one RNA bulge, 8,333,979 common off-targets were found by the two methods, 41 off-targets exclusive to GuideScan2 and 9 exclusive to CRISPRitz. When searching for off-targets with up to 5 mismatches and one DNA bulge, 1,434,370 common off-targets were found by the two methods, 62,828 off-targets exclusive to GuideScan2 and 2 exclusive to CRISPRitz. Finally, when searching for off-targets with up to 5 mismatches and one RNA and one DNA bulge, 9,614,780 common off-targets were found by the two methods, but 70,220,891 off-targets were exclusive to GuideScan2 while only 11 exclusive to CRISPRitz. This is because CRISPRitz does not find off-targets with both DNA and RNA bulges in the same off-target. A more comprehensive comparison of off-targets identified by GuideScan2, CRISPRitz, FlashFry, and Cas-OFFinder for several random samples of 100 gRNAs is presented in Table S7. The sets of identified off-targets are nearly identical between methods.

### Comparison of GuideScan2 and other web interfaces for gRNA design and analysis

The GuideScan2 web interface (https://guidescan.com) was compared with CRISPOR [56], CRISPick [28], and Cas-OFFinder [52]. The qualitative comparison of the main features, as well as comparison of selected tools by run time efficiency, is presented in Table S1.

We found that the main limitations of CRISPOR are lack of support for batch design and limited input size ($< 2300$ bp). Most importantly, CRISPOR misses off-targets and therefore underestimates specificity. For example, when searching for gRNAs in a region around TSS of CCR5 in hg38 (input coordinates chr3:46369996–46371554), the same gRNA is reported as the most specific (TATCAAGCTCTCTTGGCGGT) by both CRISPOR and GuideScan2, but GuideScan2 finds 100 off-targets with up to 4 mismatches, while CRISPOR finds only 50, despite CRISPOR looking for off-targets with PAMs NGG, NAG and NGA, while GuideScan2 only searching for NGG and NAG. Similarly GuideScan2 finds many more off-targets than CRISPOR for most other gRNAs identified for this input region. This could potentially be explained by CRISPOR relying on short-read aligner BWA for searching off-targets, while we previously demonstrated that short-read aligners such as Bowtie or BWA are suboptimal for this task and miss off-targets [5]. Another explanation, according to their manual, could be in their decision to filter out certain off-targets from display. The main limitations of CRISPick are lack of ability to design or analyze gRNAs targeting non-coding regions, limited batch design ($< 500$ inputs), no batch sequence search, no search for sequences shorter than 30 bp (e.g., individual gRNAs), and no detailed reporting of off-targets (only specificity values).

Then we ran experimental evaluation of the websites on two main features: sequence search and region search. The sequence search reports off-target information for a particular gRNA or sequence. The region search finds all gRNAs in a particular genomic region as specified by the genomic coordinates. Importantly, we found that no tool besides GuideScan2 allows for both sequence and region search at the batch level. Tools that do not enable batch queries are not suitable for large scale gRNA library design. Further, we found that most tools do not offer the ability to report complete off-target information, and only report aggregated gRNA information.

Our sequence search comparison was restricted to the only two tools that allow batch sequence search, GuideScan2 and CasOFFinder. For this experiment, we took a random set of 1000 hg38-targeting gRNAs from the aggregate set of published gRNAs reanalyzed in this study. For these gRNAs, we ran batch gRNA sequence search requesting identification of off-targets with up to 4 mismatches against the NRG PAM.

Our comparison of gRNA search by genomic coordinates was restricted to the only two tools that allow such queries in batch, GuideScan2 and CRISPRpick. For this experiment, we took a random set of 500 hg38-targeting gRNAs from the aggregate set of published gRNAs reanalyzed in this study. From this set of 500 gRNAs, we created 500 region queries using 100bp flanking regions on both sides of each gRNA.

### Generation of genome-wide gRNA databases

We used GuideScan2 to generate a collection of gRNA databases (see https://guidescan.com/) for frequently studied organisms for Cas9 and Cas12a (Cpf1) CRISPR systems. The databases are stored in a compressed BAM file format. Each row in the file corresponds to a gRNA and stores the genomic position and mismatch distance of all off-targets. Where applicable, the two scores *specificity* and *cutting efficiency* are stored in the cs and ds tags of the attributes field. Off-target information in these files is encoded in a hex format.

Database generation can be easily parallelized for efficiency. We took the following steps to parallelize the algorithm:

1. Generate the set of all 23-mers in the genome ending in NGG.
2. Partition this set of 23-mers into 1000 partitions.
3. Run GuideScan2 in parallel on each partition with PAM NGG and a secondary PAM NAG.
4. Merge the resultant SAM files together.
5. Append gRNA scores.
6. Convert SAM file to BAM format.

The database generation was performed using Memorial Sloan Kettering Cancer Center’s High Performance Computing resource. Database generation took approximately 3 days using around 1000 CPUs, though the exact timing depended upon resource contention on the cluster at the time. In step 5, we attached *cutting efficiency* score, defined using the Rule Set 2 [13], and *specificity*, as described above. This is done with a separate Python script that appends those scores to the SAM file. A complete tutorial describing the procedure for construction of genome-wide gRNA databases using GuideScan2 can be found in the software manual at https://github.com/pritykinlab/guidescan-cli [14, 15].

### Reanalysis of published CRISPRko screens

We analyzed the relationship between gRNA specificity and performance in published CRISPRko essentiality screens in human cell lines that we refer to as Sabatini2015, Doench2018 and Elling2020 [23, 24, 28]. These are genome-wide essentiality screens where each gene is targeted with a small set of gRNAs and cell fitness depletion is measured for each gRNA. Specifically, each gRNA is associated with a control and treatment sequencing read count, and from these counts fold-change values are computed. Genes with consistently strong depletion across the multiple gRNAs targeting them are thought to be essential.

We preprocessed gRNAs and converted the data into a unified CSV format. We used GuideScan2 (without off-target search) to verify the primary target locations of all gRNAs in these libraries. Human genome assembly hg38 was used as a reference. Then we ran GuideScan2 for all the gRNAs in these screens (including those not present in GuideScan2 database) against the hg38 GuideScan2 genomic index, searching for all off-targets with up to 3 mismatches while matching the alternative PAM sequence NAG.

Log2 fold change (LFC) values for the Elling2020 screens were taken from the publication [23]. LFC values for the Sabatini2015 screens were calculated from sequencing counts provided in the publication following the instructions in the publication [24]. Specifically, we filtered out any gRNAs with control counts below 400. We then normalized control and treatment counts by dividing by the total count in each column, respectively. These normalized counts were then used to compute LFC values. For the KBM7 cell line, the LFC values were averaged across the replicates. For the Doench2018 screen [28], we took the published raw counts and then normalized control and treatment counts using DESeq2 [57]. These normalized counts were then used to compute LFC values for the two replicates, and the final LFC was computed as the average across the two replicates.

To evaluate the relationship between gRNA specificity and its effect on cell fitness depletion in essentiality screens, we first classified gRNAs into two categories: those targeting essential and non-essential genes. For the Sabatini2015 data set, we chose a simple *p*-value threshold to classify genes into groups. Specifically, genes with all gRNAs with $p> 0.01$, as reported in the publication where *p*-values were calculated using a variant of the RNA interference gene enrichment algorithm (RIGER) [24], were classified as non-essential, while genes with all gRNAs with $p < 0.01$ were classified as essential. For both the Doench2018 and Elling2020 data, we used the published set of “Constitutive Core Essential Genes” and “Non Essential Genes” to classify gRNAs as described in the publication [23]. Regression between specificity and LFC was calculated using function geom_smooth() from package ggplot2 v3.3.5 in R (with default parameters). This calculation demonstrated that even for non-essential genes, gRNAs with particularly low specificity ($< 0.15$) have strongly negative LFC values that are comparable to gRNAs targeting essential genes.

We also set out to investigate how cell type-specific the gRNA effects are. To do this, we used the Sabatini2015 data since they targeted the same set of genes across four different cell lines. For each pair of cell lines, we computed the correlation between the LFC values across the same set of gRNAs and observed it to be less than 0.10 for every pair of cell lines. This suggests that gRNA cell fitness depletion effect is highly cell type-specific. Therefore, the significant relationship between our GuideScan2-derived specificity score, calculated in a cell type-agnostic manner, and cell fitness depletion is especially surprising.

### Reanalysis of published CRISPRi and CRISPRa screens

In order to investigate the effect of gRNA specificity in CRISPR inhibition and activation screens, we analyzed data from several publications herein referred to as Lim2017 [26], Weissman2016 [27], Doench2018 [28], Weissman2021 [29], and Marson2022 [30]. We first preprocessed and reanalyzed gRNAs from these screens using GuideScan2 in the same manner as for CRISPRko screens. We thus obtained off-target information and specificity scores for all gRNAs in these screens. We also obtained designations of genes as (positive or negative) hits and non-hits as defined by authors of these publications, as described below. This allowed us to compare average gRNA specificity for gRNAs targeting a gene with the designation of a gene as a hit or non-hit.

We further analyzed the Lim2017 screen [26] that aimed to detect functional long non-coding RNAs (lincRNAs). In this screen, a set of 16,401 putative lincRNA transcription start sites (TSS) were targeted with ten gRNAs per site, and log2 fold-change depletion in the screen estimated for each gRNA. Extending the analysis in the publication [26], we performed binary logistic regression classification of genes to hits and non-hits, using data combined from all the seven cell lines. Importantly, for this analysis, we used the definitions of hits and non-hits reported in the original paper. We used 80% of the data for training and 20% of the data for testing and reporting the performance. While in the publication the most predictive feature was gene expression (which is cell type-specific) [26], we discovered that the mean gRNA specificity, which is cell type-agnostic in our calculations, outperformed gene expression in predicting hits. This provides further evidence that specificity is an important confounding factor in CRISPRi screens.

We performed similar analysis for the protein coding gene essentiality CRISPRi and CRISPRa screens Weissman2016 and Doench2018 [27, 28]. For the Weissman2016 data, we reproduced the definition of screen hits and non-hits using instructions from the publication [27] (Additional File 1: Fig. S5a) and observed using Fisher’s exact test that genes with mean gRNA specificity above 0.25 were significantly enriched among hits. For this analysis, we only focused on “negative” hits. For the Doench2018 screen, we obtained the definitions of hits and non-hits from the publication [28].

For a more uniform analysis across studies, we used MAGeCK v0.5.9.5 [31] to define hits and non-hits in these screens. For each screen, we ran MAGeCK with default parameters for the same contrast as in publication (e.g., two timepoints in essentiality screens, or IL-2+ vs. IL-2– in a T cell screen) using read counts obtained from each publication, including controls. With this analysis, we classified genes into both positive and negative hits (FDR $< 0.05$). Using these definitions of hits and non-hits, the same analysis of gRNA specificities for these genes was run as above. Note that authors of the screens in T cells [30] defined as negative hits, or negative regulators of IL-2 or IFNG expression, what we defined as positive hits in the MAGeCK analysis.

### Reanalysis of genome-wide gRNA libraries

We reanalyzed previously published human and mouse genome-wide gRNA libraries from five publications [13, 18, 22–24] using GuideScan2. We refer to them as Moffat2015, Bassik2017, Root2016, Elling2020, and Sabatini2015, respectively. We observed that, consistent with previous reports, many gRNAs in these libraries had no-mismatch off-targets (i.e., multiple perfect occurrences in the genome) and overall low specificity (Additional file 1: Fig. S3c,g,h). Therefore we decided to use GuideScan2 in order to construct new genome-wide gRNA libraries with high specificity, preserving and building on the design criteria of the previous libraries.

After construction and experimental validation of the new GuideScan2 library, we compared GuideScan2 library specificities and cutting efficiency values with an extended list of genome-wide gene-targeting gRNA libraries for human and mouse that we found using https://www.addgene.org/pooled-library/. This included six additional libraries that we called Moffat2019, Parts2019, Doench2020, Garnett2021, Liu2021, and Teichmann2019 [58–63]. We still observed that GuideScan2 library specificities were the largest (Additional file 1: Fig. S3g,h).

### GuideScan2 library design

We designed new genome-wide libraries using GuideScan2 and motivated by the design criteria of previous genome-wide libraries [13, 18, 22–24]. We constructed libraries for three reference genomes, hg38, mm10, and mm39, but analogous steps could be taken for any organism of interest.

The construction followed multiple steps as described below; the numbers of gRNAs after each filtering step are provided for the hg38 genome as an example. First, we identified the potential gRNA space as the set of all 20-mer sequences followed by an NGG PAM in the genome. Then, we filtered out gRNAs that have multiple occurrences in the genome with no mismatches or one mismatch, leaving us with 160,448,682 unique gRNAs. We then further filtered out gRNAs that do not cut within a CDS region, leaving us with 4,106,943 gRNAs. Next, we filtered out gRNAs with cutting efficiency less than 0.25, leaving us with 3,964,322 gRNAs, and also gRNAs with specificity less than 0.20, leaving us with 2,329,135 gRNAs. We further removed gRNAs with extreme G/C content, defined as having the total of C and G nucleotides greater than 80% or less than 20%, leaving us with 2,289,685 gRNAs. We then excluded any gRNAs containing monopolymers of length greater than three (i.e., four or more consecutive identical nucleotides), leaving us with 2,165,665 gRNAs in hg38. These remaining 2,165,665 gRNAs were then used for our final library selection steps.

Using this filtered set of gRNAs, we attempted to find six gRNAs for each gene. For genes with six or fewer gRNAs, we used all of them. For genes with more than six gRNAs in the filtered set, we used further criteria to rank gRNAs and select six per gene. For this, we ranked gRNAs for each gene using a simple score that balances maximizing the gRNA specificity and cutting efficiency. Furthermore, the nucleotide at the 5′ end of gRNA is sometimes replaced with G for better efficiency, and therefore for each gRNA we considered specificity both for the gRNA itself and for a version of it where the nucleotide at 5′ end is replaced with a G (GuideScan2 allows to calculate both specificity values). To summarize, the score for ranking gRNAs for each gene is defined as

$$\begin{aligned} \text {Score}(g) = \min \{\text {Specificity}(g), \text {Specificity}(g'), 1.25 \cdot \text {CuttingEfficiency}(g)\}, \end{aligned}$$

where *g*’ denotes the sgRNA *g* with the nucleotide at 5′ end replaced with a G. Cutting efficiency is defined as the “Rule Set 2” score [13]. For each gene that had more than six gRNAs in the filtered list, we selected six gRNAs with the highest value of $\text {Score}(\cdot )$.

Note that multiple gRNA design and synthesis strategies have been reported in the literature to ensure G at 5′ end, resulting in gRNAs of variable length and/or with G at 5′ end that does not match DNA at the primary target location. GuideScan2 can be easily used to enumerate off-targets for any such gRNAs using the same genomic index.

Additionally, we included a set of 5000 safe-targeting control gRNAs and 5000 non-targeting control gRNAs. To generate non-targeting controls, we randomly generated gRNA sequences and selected those that had no genomic alignment with Hamming distance three or lower. For safe-targeting controls, we selected gRNAs that cut within the safe-targeting regions defined in “Supplementary Table 5” of the Bassik2017 paper [18]. As these safe-targeting regions were designed for hg19 and mm10 genome assemblies, we used LiftOver from the UCSC genome browser [64] to map the regions to hg38 and mm39, respectively. Among these safe-targeting guides, totalling 10,149,882 in hg38, we selected the 5000 with the best specificity. For both sets, we applied the same filtering steps as for our gene-targeting gRNAs where applicable.

### Design of the essentiality screen for validation of the new GuideScan2 library

In order to experimentally test the performance of our GuideScan2 genome-wide gRNA library and to confirm improved gRNA specificity without sacrificing efficiency, we designed an essentiality screen. The experiment consisted of two arms.

In the first arm, we screened a random subset of 100 essential genes as defined in a previous publication [65]. These 100 genes were selected from the top 5000 most highly expressed genes (after averaging expression values over two replicates), defined using published RNA-seq gene expression data. For these genes, we included all gRNAs from the five libraries and from the GuideScan2 library targeting these genes, resulting in 4050 gRNAs in this arm of the screen.

The second arm of the experiment aimed to demonstrate the decreased confounding effect of low-specificity gRNAs in our GuideScan2 library. For this, we selected gRNAs targeting non-essential genes. Namely, we hypothesized that low-specificity gRNAs in other libraries reduce cell fitness even when targeting non-essential genes and thus confound the interpretation of results of screens relying on these libraries. To test this, we first selected a set of non-essential genes from [66] that were poorly expressed in the A549 cell line. For all libraries except Bassik2017, we ranked genes by the average specificity of their two lowest specificity gRNAs. Then we included all gRNAs targeting the 135 top ranking (lowest specificity) genes for each library in our screen. For Bassik2017, we applied a slightly different ranking scheme as the specificity score is not defined for gRNAs of length other than 20nt. That is, we ranked genes by the average number of off-targets for the two gRNAs with the largest number of off-targets, and selected the top 135 genes. For comparison to the GuideScan2 library, we included all GuideScan2 library gRNAs targeting the same genes. This second arm of the screen totaled 6011 distinct gRNAs.

Finally, we included a random set of 100 safe-targeting and 100 non-targeting control gRNAs from the GuideScan2 library into our screen. This resulted in the total of 9115 unique gRNAs selected for the screen.

### CRISPR-Cas9 essentiality screen

#### Cloning of the CRISPR libraries

sgRNAs were divided into small pools of libraries, and oligo pools were synthesized by Agilent Technologies. To screen these libraries, we generated the pUSEBR (U6-sgRNA-EFS-Blast-P2A-TurboRFP) lentiviral vector by Gibson assembly of the following DNA fragments: (i) PCR-amplified U6-sgRNA (improved scaffold) [67] cassette, (ii) PCR-amplified EF1a promoter, (iii) PCR-amplified Blast-P2A-TurboRFP gene fragment (IDT), and (iv) BsrGI/PmeI-digested pLL3-based lentiviral backbone [68]. Libraries were cloned into pUSEBR using a modified version of the protocol published by Doench et al. [13] to ensure a library representation of > 10,000-fold. Briefly, each library was selectively amplified using uniquely barcoded forward and reverse primers that append cloning adapters at the 5′ and 3′ ends of the sgRNA insert, purified using the QIAquick PCR Purification Kit (Qiagen), and ligated into BsmBI/Esp3I-digested and dephosphorylated pUSEBR, using high-concentration T4 DNA ligase (NEB). A total of 1.2 $\upmu$g of ligated pUSEBR-CRISPR Library plasmid DNA was then electroporated into Endura electrocompetent cells (Lucigen). Competent cells were recovered for 1 h at 37 °C, plated across four 15-cm LB-Carbenicillin plates (Teknova), and incubated at 37 °C for 16 h. The total number of bacterial colonies per sub-pool was quantified using serial dilution plates, to ensure a library representation of > 10,000-fold. The next morning, bacterial colonies were scraped and briefly expanded for 4 h at 37 °C in 500 mL of LB-Carbenicillin. Plasmid DNA was isolated using the QIAfilter Plasmid Maxi Kit (Qiagen). To assess sgRNA distribution, the sgRNA target region was amplified using primers that append Illumina sequencing adapters on the 5′ and 3′ ends of the amplicon, as well as a random nucleotide stagger and unique demultiplexing barcode on the 5′ end. Library amplicons were size-selected on a 2% agarose gel, purified using the QIAquick Gel Extraction Kit (Qiagen), and sequenced on an Illumina NextSeq instrument (75nt single-end reads).

#### CRISPR-Cas9 screening

A549 cells expanded from a single clone harboring a doxycycline-inducible Cas9 (Addgene #114010) were a gift from J.T. Poirier [34]. To ensure that most cells harbor a single sgRNA integration event, the volume of viral supernatant that would achieve an MOI of $\approx 0.1$ upon transduction was determined. Briefly, cells were plated at a density of $0.5 \times 10^6$ cells per well in 12-well plates along with increasing volumes of a 1 to 10 dilution of the master pool viral supernatant and polybrene (EMD Millipore, 8 $\upmu$g/mL). Cells were then incubated at 37 °C overnight. Viral infection efficiency was determined by the number of surviving blasticidin-resistant (Gibco, 10 $\upmu$g/mL) cells as compared to unselected control assessed using Countess II FL (Thermo Fisher). The volume of viral supernatant that achieve 10% infection rate was used in the screen. To ensure a representation of 3250X at the transduction step, the appropriate number of cells were transfected with viral supernatant in 150 mm tissue culture plates (Corning) per infection replicate and selected with blasticidin for 5 days. Subsequently, half of the blasticidin-selected cells were pelleted and stored at – 80 °C (cumulative population doubling T0/input population) while the rest were plated and treated with doxycycline (Millipore-Sigma, 1 $\upmu$g/mL) for 18 days, or approximately 18 population doublings (TF/Final). At least 200 million cells were harvested and pelleted for this final time point.

#### Genomic DNA isolation

Genomic DNA was extracted from cells using phenol-chloroform extraction. Briefly, cell pellets were incubated at 50 °C overnight in lysis buffer (100mM Tris-HCl pH 8.5, 200mM NaCl, 5mM EDTA, 0.2% SDS) containing 200 $\upmu$g/mL proteinase K (Roche). After digestion, the supernatant was first extracted using phenol:chloroform:isoamyl alcohol 25:24:1 pH 8.0 (Millipore-Sigma), followed by chloroform:isoamyl alcohol 49:1 (Millipore-Sigma). The gDNA was then precipitated with equal volume of 100% isopropanol, washed with 70% ethanol, and resuspended in UltraPure distilled water (Gibco).

#### Library amplification of CRISPR screens

Libraries were amplified from gDNA by a modified 2-step PCR version of the protocol published by Doench et al. [13]. Briefly, an initial “enrichment” PCR was performed, whereby the integrated sgRNA cassettes were amplified from gDNA (PCR#1), followed by a second PCR to append Illumina sequencing adapters on the 5′ and 3′ ends of the amplicon, as well as a random nucleotide stagger and unique demultiplexing barcode on the 5′ end (PCR#2). Each “PCR#1” reaction contained 25 $\upmu$L of Q5 High-Fidelity 2X Master Mix (NEB), 2.5 $\upmu$L of Nuc PCR#1 Fwd Primer (10 $\upmu$M), 2.5 $\upmu$L of Nuc PCR#1 Rev Primer (10 $\upmu$M), and 5 $\upmu$g of gDNA in 20 $\upmu$L of water. PCR#1 amplicons were selected on a 2% agarose gel and purified using the QIAquick Gel Extraction Kit (Qiagen). These amplicons were then used as template for “PCR#2” reactions. Each PCR#2 reaction contained 25 $\upmu$L of Q5 High-Fidelity 2X Master Mix (NEB), 2.5 $\upmu$L of a unique Nuc PCR#2 Fwd Primer (10 $\upmu$M), 2.5 $\upmu$L of Nuc PCR#2 Rev Primer (10 $\upmu$M), and 300 ng of PCR#1 product in 20 $\upmu$L of water. Library amplicons were size-selected on a 2% agarose gel, purified using the QIAquick Gel Extraction Kit (Qiagen), and sequenced on an Illumina NextSeq500 instrument (75 nt single end reads). PCR settings for PCR#1 and PCR#2 were: initial denaturation at 98 °C for 30 s; then 98 °C for 10 s, 65°C for 30 s, 72 °C for 30 s for 24 cycles; followed by extension at 72 °C for 2 min.

#### Analysis of CRISPR-Cas9 screen data

FASTQ files were processed and aligned to the reference sgRNA library file, and log2 fold change value for each sgRNA was calculated using MAGeCK v0.5.9.2 [31]. Only the gRNAs with control read count > 100 were used in subsequent analysis. For analysis of gRNAs targeting essential genes, average LFC per gene was calculated across all gRNAs used in the screen for that gene, and only the genes with such average below $-1$ were used, resulting in a selection of 85 genes out of 100 genes. The remaining analysis for both essential and non-essential genes followed in a straightforward way from the design of the screen.

#### Comparison of results for essential genes

Furthermore, we separately compared efficiency of the libraries for essential genes. Namely, our screen contained all gRNAs from six libraries for 100 randomly selected essential genes. In order to assess the significance of negative LFC values for each gene from each library in our screen, we performed a one-sided Wilcoxon signed rank test. From 100 genes, at $p < 0.1$, 54 genes had significantly negative LFC for library Root2016, 77 genes for Sabatini2015, 80 genes for Elling2020, 60 genes for Moffat2015, 76 genes for Bassik2017, as compared to 83 genes for GuideScan2 library. Note that all libraries except Root2016 had 6 or more gRNAs per gene, while Root2016 had 4 gRNAs per gene. In order to make a more appropriate comparison against the Root2016 library, we randomly selected 4 out of 6 gRNAs for each gene in the GuideScan2 library and performed the same analysis, repeating it 100 times; this resulted in an average of 61.9 significant genes (standard deviation 2.3), which is still larger than for Root2016. This analysis confirmed that the GuideScan2 library was more efficient than other libraries for targeting essential genes.

### Design of the genome-wide gRNA database for allele-specific editing

We used GuideScan2 to design a set of allele-specific gRNAs for an F1 cross between C57BL/6 and 129S1/SvlmJ mouse strains, thereafter referred to as B6 and 129S1.

First, we constructed a synthetic 129S1 pseudo-genome using MMARGE v1.0 [69] by introducing sequence variants obtained from the Mouse Genome Project (files 129S1_SvImJ.mgp.v5.snps.dbSNP142.vcf and 129S1_SvImJ.mgp.v5.indels.dbSNP142.normed.vcf obtained from ftp://ftp-mouse.sanger.ac.uk/REL-1505-SNPs_Indels/strain_specific_vcfs) [70] to the reference B6 genome assembly mm38 (file GRCm38_68.fa). Then we constructed the GuideScan2 gRNA database for this synthetic 129S1 pseudo-genome, as well as separately for the B6 genome.

For each gRNA from the 129S1 database, we used GuideScan2 to search for it in the B6 index and filtered it out if a perfect occurrence was found; we also performed a reverse analysis and filtering of B6 gRNAs against the 129S1 index. The remaining gRNAs after this filtering formed the list of allele-specific gRNAs. We then used GuideScan2 to search for off-targets of these gRNAs in both 129S1 pseudo-genome and B6 genome.

The allele-specific gRNAs were annotated with protein-coding genes from GENCODE vM29 (file gencode.vM29.annotation.gtf.gz). A gRNA was annotated with a gene if the corresponding cutting site (3nt upstream of PAM) was within an exon of that gene. If multiple such gene annotations satisfied this criterion, an arbitrary one of them was chosen. Of the 21,812 protein-coding genes, 8079 had a B6-specific gRNA, and 8330 had a 129S1-specific annotation, while 7187 genes had both.

### Experimental validation of allele-specific gRNAs

#### Selection of gRNAs for validation

For experimental validation of the gRNA database for allele-specific targeting in F1 B6/129S1 cross described above, a representative set of ten gene-targeting gRNAs were manually selected (Table S6). Furthermore, we used an additional set of gRNAs randomly sampled from a pre-selected pool of allele-specific gRNAs of higher confidence. Namely, for the selection of this random gRNA sample, of all 5M presumably allele-specific gRNAs in the database (Table S5), we filtered out those annotated with heterozygous variants (yielding 4.9M gRNAs). Then we selected only gRNAs without any 2-mismatch off-targets in either B6 genome or 129S1 pseudo-genome, resulting in 1.2M gRNAs. From those, we selected only gRNAs with at most three 3-mismatch off-targets in either genome, resulting in 141.8K gRNAs. Among those, we selected the gRNAs with cutting efficiency in the top 25% (or cutting efficiency $> 0.604$), resulting in 35.4K gRNAs. Finally, we filtered out gRNAs within 200bp of annotated repetitive regions (repeat masker file mm10_rmsk.bed downloaded from the UCSC Genome Browser), resulting in the final list of 12,932 gRNAs. From this final list, we selected ten B6-specific and ten 129S1-specific gRNAs at random (Table S6). Most of them appeared to be targeting genomic regions outside of genes. crRNA, according to the differential sgRNA spacer sequences, were synthesized by IDT.

#### CRISPR-Cas9 editing

V6.5 mouse embryonic stem cells (gift from Rudolf Jaenisch), which was generated from a F1 cross between C57BL/6 and 129S1/SvlmJ mouse strains, were plated in 6-well plates at a density of $0.5 \times 10^6$ cells per well 24 hours prior to transfection. Alt-R CRISPR-Cas9 crRNAs with spacer sequences that would differentially target the C57BL/6 and 129S1/SvlmJ alleles, as well as a control spacer that would not differentially target the C57BL/6 and 129S1/SvlmJ alleles, were synthesized by IDT and were annealed with Alt-R CRISPR-Cas9 tracrRNA ATTO 550 (IDT) according to manufacturer’s protocol. Following, Alt-R S.p. Cas9 nuclease V3 (IDT) was mixed with the crRNA-tracrRNA duplex and transfected into V6.5 mESCs using Lipofectamine CRISPRMAX (Thermo Fisher) according to the manufacturer’s protocol. Transfection efficiency was determined by ATTO 550 fluorescence after 24 h, as visualized by the Axio Observer A1 inverted microscope (Zeiss). Cells were then collected at 72 h post transfection and genomic DNA was extracted using phenol-chloroform as previously described above.

#### CRISPR sequencing

Edited gDNA sequences were amplified using Q5 polymerase (NEB) using specific PCR primer pairs that flank an approximately 250bp region encompassing the CRISPR-Cas9 cut site. Each reaction contained 10 $\upmu$L of Q5 Reaction Mix (NEB), 10 $\upmu$L of Q5 GC Enhancer Buffer (NEB), 2.5 $\upmu$L of a forward primer (10 $\upmu$M), 2.5 $\upmu$L of a reverse primer (10 $\upmu$M), 1 $\upmu$L of dNTPs (10mM), 0.5 $\upmu$L of Q5 polymerase (NEB), and 1 $\upmu$g of gDNA in 23.5 $\upmu$L of water. PCR settings were: initial denaturation at 98 °C for 3 min; then 98°C for 10 s, 65 °C for 30 s, 72 °C for 30 s for 25 cycles; followed by extension at 72 °C for 2 min. PCR amplicons were size selected on a 2% agarose gel and purified using the QIAquick Gel Extraction Kit (Qiagen), and sequenced using the Amplicon-EZ service provided by GENEWIZ/AZENTA (South Plainfield, NJ).

#### Analysis of CRISPR-Cas9 editing data

FASTQ files provided by GENEWIZ/AZENTA were analyzed for allele-specific editing using CRISPResso2 v2.2.7 [71]. First, unmodified amplicons corresponding to either the C57BL/6 or the 129S1/SvlmJ allele and the spacer sequence were provided to CRISPResso2. Reads were then assigned as either modified or unmodified for each allele, or ambiguous. Ambiguous and unmodified reads were discarded. Cumulative fractions of modified reads attributed to C57BL/6 or 129S1/SvlmJ were calculated and tabulated for each unique spacer sequence.

## Supplementary Information

Supplementary Material 1: Supplementary Tables. Table S1: Quantitative and qualitative comparisons of the GuideScan2 algorithm to existing software for gRNA design and analysis. Table S2: Parameters of GuideScan2 and other published genome-wide gene-targeting gRNA libraries. Table S3: GuideScan2 genome-wide gene-targeting gRNA libraries for mouse and human. Table S4: Design and results of the validation gene essentiality screen of the human GuideScan2 gene-targeting library. Table S5: Genome-wide gRNA library for allele-specific targeting in F1 C57BL/6 $\times$ 129S1/SvlmJ hybrid mice. Table S6: Design and results of the allele-specific targeting validation experiment. Table S7: Comparison of off-targets identified by CRISPR gRNA analysis methods.

Supplementary Material 2: Additional File 1: Supplementary text containing details of the GuideScan2 algorithm along with Supplementary Figures S1–S9

## Data Availability

All processed data reported in this work, as well as reanalyzed data from previous publications, is provided in Supplementary Tables S1–S7. New sequencing data were deposited at GEO with accession number GSE285667 [72]. Software has been released on GitHub [14, 25] under the MIT license and archived on Zenodo [15].
