# Supplementary material for: Genome-wide CRISPR guide RNA design and specificity analysis with GuideScan2
Source: Genome Biol. 2025 Feb 26;26:41. doi: 10.1186/s13059-025-03488-8 (PMC11863968; doi:10.1186/s13059-025-03488-8)
Supplement: Supplementary file 2 — Supplementary Material 2: Additional File 1: Supplementary text containing details of the GuideScan2 algorithm along with Supplementary Figures S1–S9 [file 13059_2025_3488_MOESM2_ESM.pdf]

Additional File 1: Supplementary text containing details of the GuideScan2 algorithm along with Supplementary Figures S1–S9

Contents

1 Supplementary Note: Details of the GuideScan2 Algorithm 1

1.1 Computational Problem Statement 1

1.2 The GuideScan2 Algorithm 2

1.3 Extension to RNA and DNA Bulges 6

1.4 Time Saving Heuristics 6

2 Supplementary Figures 10

1 Supplementary Note: Details of the GuideScan2 Algorithm

1.1 Computational Problem Statement

The off-target enumeration problem is to find all off-target loci that a guide RNA can recognize within a specified CRISPR system and genome. The solution depends both on the genome and the rule for classifying a genomic locus as an off-target; both are defined below.

Let us fix a finite alphabet Σ and let Σ^* be the set of all finite strings with characters in Σ. The length of a string $s \in \Sigma^*$ is written as $|s|$. The ith character in $s \in \Sigma^*$ is denoted as s_i, starting with $i = 1$. A substring of $s \in \Sigma^*$ that starts at position i and ends at position j is denoted $s[i, ..., j] = s_i \dots s_j$. The *Hamming distance* between two length n strings $s = s_1 s_2 \dots s_n$ and $t = t_1 t_2 \dots t_n$ is $|\{i : s_i \neq t_i\}|$.

Using this notation, we denote a guide RNA as $g \in \Sigma^*$ and a genome as $S \in \Sigma^*$. Provided both a guide RNA g and a genome S, we define an off-target to be any substring of S of length $|g|$ that is of small Hamming distance from g. Formally, GuideScan2 solves the following string search problem, where we use the more general terms *query* and *reference* for guide RNA and genome respectively.

Problem 1. *Provided a query $q \in \Sigma^*$, a reference $S \in \Sigma^*$, and a parameter k, the* string-search problem *is to find all length $|q|$ substrings of S that are within a Hamming distance of k from q.*

The input to the problem is a query q and a reference S, but we will assume throughout that the reference S is fixed. Although the assumption that S is fixed is not amenable to all scenarios, it holds in CRISPR screen design and analysis tasks where the genome changes infrequently. Another variant of the problem is to allow r_1 deletions in the query string q, enabling us to model RNA bulges.

Definition 1. *A string s is a (k, r_1)-*off-target *of q if $|s| = |q| - r_1$ and there exists exactly r_1 deletions of characters in q such that the resultant string is of Hamming distance at most k from s.*

Symmetrically, we can allow up to r_2 deletions in the reference string to model DNA bulges, leading to the following definition.

Definition 2. *Strings s and q are (k, r_1, r_2)-*off-targets *if there exists at most r_1 deletions from q and at most r_2 deletions from s such that the Hamming distance of the resultant strings is at most k.*

A generalized version of the string search problem is to enumerate all (k, r_1, r_2)-off-targets. We show how to extend our solution to the string-search problem to this more general setting in Section 1.3.

Problem 2. *Provided a query $q \in \Sigma^*$, a reference $S \in \Sigma^*$, and a parameter (k, r_1, r_2), the* generalized string-search problem *is to find all substrings s of S that are (k, r_1, r_2)-off-targets with q.*

1.2 The GuideScan2 Algorithm

GuideScan2 uses a simulated trie traversal algorithm to search for off-targets. Rather than keep a full trie in memory, which is prohibitively large, it uses the Burrows-Wheeler Transform (BWT) [1] to implicitly reference a trie structure.

To solve the string-search problem and introduce the GuideScan2 algorithm, we first review suffix tries [2], trees that allow linear time search of any substring by pre-processing all suffixes.

Definition 3. *A suffix trie over a string $S \in \Sigma^n$ is a directed, rooted tree $\mathcal{T} = (V(\mathcal{T}), E(\mathcal{T}))$ satisfying four properties:*

- **(P1):** *Each edge is labeled by a character $\sigma \in \Sigma$.*
- **(P2):** *No two out-edges of a node $v \in \mathcal{T}$ are labeled by the same character.*
- **(P3):** *Every suffix $S[k, \ldots, n]$, $1 \leq k \leq n$, is labeled by some leaf $v \in V(\mathcal{T})$ and every leaf is labeled by some suffix.*
- **(P4):** *Each node v is labeled by the string $s(v) := s_1 s_2 \ldots s_k$ where s_i is the character on the ith edge on the path from root to v.*

Observe that *i)* every substring $S[k, \ldots, l]$ is a length $(l - k + 1)$ prefix of the suffix $S[k, \ldots, n]$ and *ii)* every path from the root to a node v is a prefix of some suffix **(P3)**. Thus, to determine if q is a substring of S, it suffices to walk down from the root of our suffix trie until we have matched all characters in q.

Using the observation above, we obtain an algorithm for the *string search-problem* provided we have already constructed a suffix trie for S. Specifically, we perform a depth-first search (DFS) through the trie to find all substrings of S of Hamming distance at most k from the query q. We start the DFS at the root node with a cost c and index i initialized to 0 and 1 respectively. At each node v, we follow all out-edges if $c < k$ and increment our string index i by 1. If $c = k$, we only follow out edges matching the character at the index i. If the followed out-edge does not match the character at index i, we increment c by 1, if it does match, we do not increment c. Once we have reached the end of our query string q, we emit the matching string. In the case where $c = k$ and there are no out-edges matching the index, we terminate without emission. This algorithm is described formally as **Algorithm 1**. By an inductive argument on prefixes of q, one can observe that it solves the *string-search problem.*

Algorithm 1 GuideScan2 Algorithm Using a Trie

Require: A suffix trie $\mathcal{T}$ over S, a query string q, a parameter k, an index i and cost c initialized to 0, and a node u initialized at root.

if $i - 1 = |q|$ **then**
    emit u
    return
end if

for $v \in \text{children}(u)$ **do**
    if $\text{label}(u, v) = q_i$ **then**
        recurse with ($\mathcal{T}$, k, q, $i + 1$, c, v)
    else if $c < k$ **then**
        recurse with ($\mathcal{T}$, k, q, $i + 1$, $c + 1$, v)
    end if
end for

Theorem 1. *The suffix-trie search algorithm described in* **Algorithm 1** *solves the string-search problem in* $\mathcal{O}\left(\binom{|q|}{k}(|\Sigma| - 1)^k\right)$ *time.*

Though the solution to the *string-search problem* takes time exponential in $|q|$ and k, it is of interest that it does not depend on the length of S. This allows the approach to scale up to large reference strings.

It is not quite the case that we can directly view GuideScan2's search algorithm as over a suffix trie. However, it can be viewed as a search over a related structure, that of a reverse prefix trie [3]. A reverse prefix trie is identical to that of a suffix trie but instead of inserting each suffix into the trie, we insert the reverse of every prefix, $S[1, \ldots, k]$, into the trie.

Definition 4. *A reverse prefix trie over a string* $S \in \Sigma^n$ *is a directed, rooted tree* $\mathcal{T} = (V(\mathcal{T}), E(\mathcal{T}))$ *satisfying four properties:*

- **(Q1):** *Each edge is labeled by a character* $\sigma \in \Sigma$.
- **(Q2):** *No two out-edges of a node* $v \in \mathcal{T}$ *are labeled by the same character.*
- **(Q3):** *The reverse of every prefix* $S[1, \ldots, k]$, $1 \leq k \leq n$ *is labeled by some leaf* $v \in V(\mathcal{T})$ *and every leaf is labeled by the reverse of some prefix.*
- **(Q4):** *Each node* v *is labeled by the string* $s(v) := s_k s_{k-1} \ldots s_1$ *where* s_i *is the label of the* i^{th} *edge on the path from root to* v.

Symmetric to the fact that every substring is the prefix of some suffix, every substring is the suffix of some prefix. Thus, we can use the reverse prefix trie in an identical manner as the suffix trie by matching our query string q, *albeit in reverse.* Many algorithms on suffix tries can be viewed as algorithms over reverse prefix tries by simply reversing the query string [3].

Now, we introduce a labeling scheme to the nodes within a reverse prefix trie such that we can simulate a walk down the trie by enumerating the labels of a node's children. Specifically, let $S' = S\$$ be the string resulting from appending a character $\$ \notin \Sigma$ to S, and consider the lexicographically sorted list of all $n + 1$ cyclical permutations of S', $L = (S'_0, S'_1, \ldots, S'_n)$. We will assume that the introduced \$ character compares

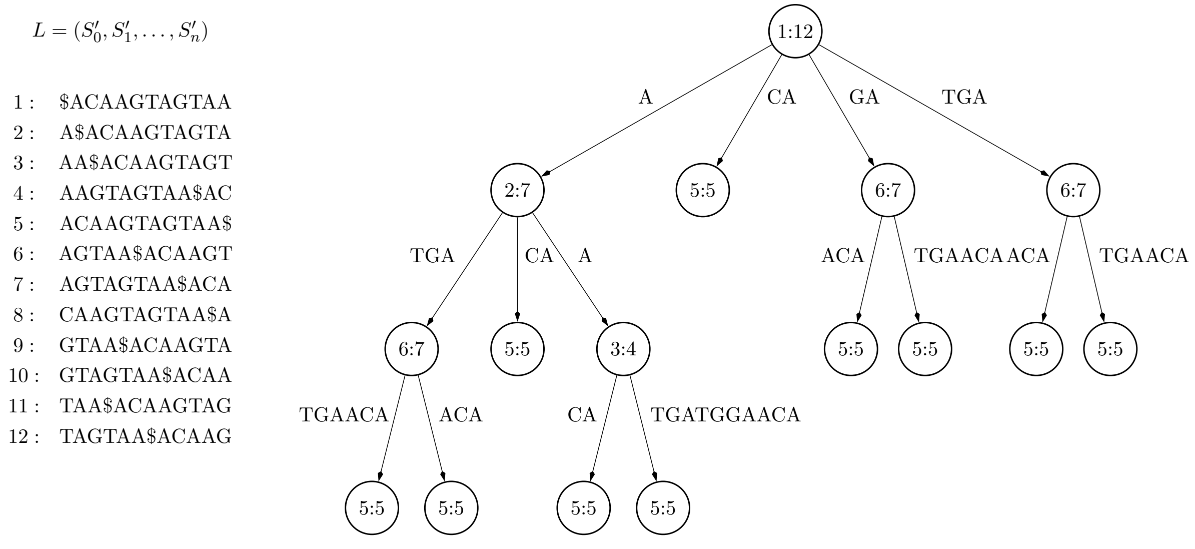

Figure 1: The reverse prefix trie for the string $S = \text{ACAAGTAGTAA}$ and the list L of sorted cycles. We contract degree 2 nodes in $\mathcal{T}$ to simplify the diagram.

less than every other character so the list L corresponds to lexicographically sorting the suffixes of S. Our labeling scheme $\mu(v)$ consists of the indices of the strings in L that have the reverse of $s(v)$ as a prefix. Said another way, $\mu(v)$ is the set of rows of L, when viewed as a matrix, match $s(v)$. Formally, we write

$$\mu(v) := \{i \in [n] : S'_i \text{ has reverse prefix } s(v)\}.$$

Since we sorted the strings in L lexicographically, one can observe that $\mu(v)$ consists of an interval. Specifically, if $i, j \in \mu(v)$ and $i \leq j$, $k \in \mu(v)$ for all $k \in [i, j]$ since if S'_i and S'_j start with the reverse of $s(v)$, S'_k must also start with the reverse of $s(v)$. Thus we can describe $\mu(v)$ by two integers and write $\mu(v) = [\mu_0(v), \mu_1(v)]$. An example of the labeling scheme for the reverse prefix trie can be found in **Figure 1**.

This labeling μ of the reverse prefix trie is useful for several reasons. First, since L consists of all sorted cycles, the length of the interval $\mu(v)$ corresponds to the number of substrings of the reverse of $s(v)$ in S. Second, the length of the interval $\mu(v)$ also corresponds to the number of leaves in the sub-tree rooted at v. Third, if we have access to L, we can enumerate the location of each substring $s(v)$. Thus, for the purpose of finding matching strings, $\mu(v)$ is sufficient. Finally, with only knowledge of $\mu(v)$, we show that we can compute $\mu(u)$ for all children u of v labeled and also use $\mu(v)$ to determine if a child u exists.

Taken together, we simulate our walk down the reverse prefix trie by evaluating μ to determine the children to which we walk. The trick of the Burrows-Wheeler Transform is that we can evaluate μ at a child quickly given μ at the parent node [4, 5, 6, 7]. To do this we will introduce the Burrows-Wheeler Transform (BWT) [1] which is defined as

$$\text{BWT}(S) = S'_1[n]S'_2[n] \dots S'_n[n].$$

We will also need a function $C : \Sigma \to \mathbb{N}$ that stores the first index i of a character σ in the string $S'_1[1]S'_2[1] \dots S'_n[1]$. And finally, a function that counts the number of occurrences of a character σ before an index i in the BWT of S:

$$\text{Occ}(\sigma, i) = |\{j \in [i-1] : \text{BWT}(S)[j] = \sigma\}|.$$

Algorithm 2 GuideScan2 Algorithm Using Occ(·) Oracle

Require: A function Occ(·) over S, a query string q, a parameter k, an index i and cost c initialized to 0, and a label (μ_0, μ_1) initialized to $(1, |S|)$.

```
if i + 1 = |q| then
    emit (μ_0, μ_1)
    return
end if

μ'_0 ← C(q[i]) + Occ(q[i], μ_0)
μ'_1 ← C(q[i]) + Occ(q[i], μ_1 + 1) − 1

if μ'_1 ≥ μ'_0 then
    recurse with (Occ(·), k, q, i + 1, c, (μ'_0, μ'_1))
end if

if c ≥ k then return end if

for σ ∈ Σ − {q[i]} do
    μ'_0 ← C(σ) + Occ(σ, μ_0)
    μ'_1 ← C(σ) + Occ(σ, μ_1 + 1) − 1
    if μ'_1 ≥ μ'_0 then
        recurse with (Occ(·), k, q, i + 1, c + 1, (μ'_0, μ'_1))
    end if
end for
```

We have the following relationship between the μ-labeling of a parent and its child [4, 6].

Proposition 1. *If $Occ(\sigma, \mu_1(v) + 1) - Occ(\sigma, \mu_0(v)) > 0$ then there is a (v, u) edge in the reverse prefix trie of S labeled by σ and*

$$\mu_0(u) = C(\sigma) + Occ(\sigma, \mu_0(v))$$
$$\mu_1(v) = C(\sigma) + Occ(\sigma, \mu_1(v) + 1) - 1.$$

Otherwise, there is no out-edge of v labeled by a character σ.

Consequently, provided oracles for Occ(·) and $C(\cdot)$, one is able to simulate a walk down the reverse prefix trie $\mathcal{T}$ without needing to have constructed $\mathcal{T}$. This proposition then directly yields an algorithm for solving the string-search problem that is detailed in **Algorithm 2**.

Theorem 2. *Given that $Occ(\sigma, i)$ takes $\mathcal{O}(f(n))$ time to compute,* **Algorithm 2** *solves the string-search problem in $\mathcal{O}\left(\binom{|q|}{k}(|\Sigma| - 1)^k f(n)\right)$ time.*

The time-complexity is the same (up to an $f(n)$ factor) as using a suffix-trie directly but we no longer need to store the trie in memory. This is because $C(\cdot)$ is easy to compute and Occ(·) can be quickly computed using succinct data structures that require only a small amount of memory. An efficient approach for

representing and computing Occ(·) is provided via Wavelet Trees of which several implementations are conveniently provided by the SDSL library [8]. In our implementation, $f(n) = \log|\Sigma|$ and typically, $|\Sigma| \leq 10$.

1.3 Extension to RNA and DNA Bulges

The extension of **Algorithm 2** to RNA-bulges and DNA-bulges (Problem 2) is quite natural when considering the problem as a trie-traversal. Specifically, we keep counters for the number of RNA and DNA bulges, named c_R and c_D, at each step of our depth-first search. To create an RNA bulge, we consume a character of our string without traversing down the trie. To create a DNA bulge, we walk one step down our trie without consuming a character. We update the values of c_R and c_D correspondingly. We stop creating RNA bulges when c_R reaches r_1 and stop creating DNA bulges when c_D reaches r_2. This strategy is detailed as **Algorithm 3**.

From the above description, it should be clear that **Algorithm 3** is correct since all bulges are possible and any off-target with a bulge will be matched. Since there are $|q|$ places for RNA and DNA bulges to occur, the time complexity is $\mathcal{O}\left(|q|^{r_1+r_2}\binom{|q|}{k}(|\Sigma|-1)^k\right)$. These two observations yield the following theorem.

Theorem 3. *Given that $Occ(\sigma, i)$ takes $\mathcal{O}(f(n))$ time to compute,* **Algorithm 3** *solves the generalized string-search problem in $\mathcal{O}\left(|q|^{r_1+r_2}\binom{|q|}{k}(|\Sigma|-1)^k f(n)\right)$ time.*

This completes our description of the GuideScan2 algorithm.

1.4 Time Saving Heuristics

There are several ways to require that off-targets have a matching protospacer adjacent motif (PAM). For example, one could append the PAM to their query string and require that all off-targets match perfectly in the suffix containing the PAM. This would require repeating the entire search for all PAMs, incurring more cost than necessary. Instead, we first find candidate off-targets without PAMs and then filter for the PAM among these candidate off-targets.

Because of the exponential blowup in search space even for small values of k, two heuristics are employed to speed up the algorithm's performance. First, since our simulated reverse prefix trie traversal searches for strings in reverse order, the PAM would be searched at the beginning of our string. As PAMs often contain the wildcard character N, e.g. the canonical NGG PAM for Cas9, this would require a total of four branchings before the search space had been substantially pruned. To alleviate the issue, we instead search for the reverse complement of the guide RNA on the reverse complement strand so that the PAM validation is done at the end of the search. This leads to an approximately four-fold performance improvement versus the naive strategy that searches for the forward guide on the forward strand, as the branching on N is done at the end when the search space has already been significantly pruned. Second, by default we short circuit when we find guide RNAs with multiple perfect matches since these guide RNAs are unsuitable for guide design.

Algorithm 3 GuideScan2 Algorithm with RNA and DNA Bulges Using Occ($\cdot$) Oracle

Require: A function Occ($\cdot$) over S, a reverse query string s, parametsr (k, r_1, r_2), a cost c initialized to 0, a count c_R initialized to 0, a count c_D initialized to 0, and a label (μ_0, μ_1) initialized to $(1, |S|)$.

if $i + 1 = |q|$ **then**
    emit (μ_0, μ_1)
    return
end if

$\mu'_0 \leftarrow C(q[i]) + \text{Occ}(q[i], \mu_0)$
$\mu'_1 \leftarrow C(q[i]) + \text{Occ}(q[i], \mu_1 + 1) - 1$

if $\mu'_1 \geq \mu'_0$ **then**
    recurse with $(\text{Occ}(\cdot), k, q, i+1, c, c_R, c_D, (\mu'_0, \mu'_1))$
end if

if $c \geq k$ **then return end if**

for $\sigma \in \Sigma - \{q[i]\}$ **do**
    $\mu'_0 \leftarrow C(\sigma) + \text{Occ}(\sigma, \mu_0)$
    $\mu'_1 \leftarrow C(\sigma) + \text{Occ}(\sigma, \mu_1 + 1) - 1$
    if $\mu'_1 \geq \mu'_0$ **then**
        recurse with $(\text{Occ}(\cdot), k, q, i+1, c+1, c_R, c_D, (\mu'_0, \mu'_1))$
    end if
end for

if $c_R \geq r_1$ **then return end if**

recurse with $(\text{Occ}(\cdot), k, q, i+1, c, c_R + 1, c_D, (\mu_0, \mu_1))$

if $c_D \geq r_2$ **then return end if**

for $\sigma \in \Sigma$ **do**
    $\mu'_0 \leftarrow C(\sigma) + \text{Occ}(\sigma, \mu_0)$
    $\mu'_1 \leftarrow C(\sigma) + \text{Occ}(\sigma, \mu_1 + 1) - 1$
    if $\mu'_1 \geq \mu'_0$ **then**
        recurse with $(\text{Occ}(\cdot), k, q, i, c, c_R, c_D + 1, (\mu'_0, \mu'_1))$
    end if
end for

References

[1] Michael Burrows and David J Wheeler. A block-sorting lossless data compression algorithm. 1994.

[2] Peter Weiner. Linear pattern matching algorithms. In *14th Annual Symposium on Switching and Automata Theory (swat 1973)*, pages 1–11. IEEE, 1973.

[3] Jouni Sirén. Fm-index and the reverse prefix trie.

[4] Edward Fernandez, Walid Najjar, and Stefano Lonardi. String matching in hardware using the fm-index. In *2011 IEEE 19th Annual International Symposium on Field-Programmable Custom Computing Machines*, pages 218–225. IEEE, 2011.

[5] Heng Li and Richard Durbin. Fast and accurate short read alignment with burrows–wheeler transform. *Bioinformatics*, 25(14):1754–1760, 2009.

[6] Giovanni Manzini. An analysis of the Burrows–Wheeler transform. *Journal of the ACM (JACM)*, 48(3):407–430, 2001.

[7] Ben Langmead and Steven L Salzberg. Fast gapped-read alignment with Bowtie 2. *Nature Methods*, 9(4):357, 2012.

[8] Simon Gog, Timo Beller, Alistair Moffat, and Matthias Petri. From theory to practice: Plug and play with succinct data structures. In *13th International Symposium on Experimental Algorithms, (SEA 2014)*, pages 326–337, 2014.

[9] Jonathan L Schmid-Burgk, Linyi Gao, David Li, Zachary Gardner, Jonathan Strecker, Blake Lash, and Feng Zhang. Highly parallel profiling of Cas9 variant specificity. *Molecular cell*, 78(4):794–800, 2020.

[10] John G Doench, Nicolo Fusi, Meagan Sullender, Mudra Hegde, Emma W Vaimberg, Katherine F Donovan, Ian Smith, Zuzana Tothova, Craig Wilen, Robert Orchard, Herbert W Virgin, Jennifer Listgarten, and David E Root. Optimized sgRNA design to maximize activity and minimize off-target effects of CRISPR-Cas9. *Nature Biotechnology*, 34(2):184–191, 2016.

[11] Patrick D Hsu, David A Scott, Joshua A Weinstein, F Ann Ran, Silvana Konermann, Vineeta Agarwala, Yinqing Li, Eli J Fine, Xuebing Wu, Ophir Shalem, et al. DNA targeting specificity of RNA-guided Cas9 nucleases. *Nature Biotechnology*, 31(9):827–832, 2013.

[12] Kendall R. Sanson, Ruth E. Hanna, Mudra Hegde, Katherine F. Donovan, Christine Strand, Meagan E. Sullender, Emma W. Vaimberg, Amy Goodale, David E. Root, Federica Piccioni, and John G. Doench. Optimized libraries for CRISPR-Cas9 genetic screens with multiple modalities. *Nature Communications*, 9(1):5416, 2018.

[13] S. John Liu, Max A. Horlbeck, Seung Woo Cho, Harjus S. Birk, Martina Malatesta, Daniel He, Frank J. Attenello, Jacqueline E. Villalta, Min Y. Cho, Yuwen Chen, Mohammad A. Mandegar, Michael P. Olvera, Luke A. Gilbert, Bruce R. Conklin, Howard Y. Chang, Jonathan S. Weissman, and Daniel A. Lim. CRISPRi-based genome-scale identification of functional long noncoding RNA loci in human cells. *Science*, 355(6320):eaah7111, 2017.

[14] James K Nuñez, Jin Chen, Greg C Pommier, J Zachery Cogan, Joseph M Replogle, Carmen Adriaens, Gokul N Ramadoss, Quanming Shi, King L Hung, Avi J Samelson, et al. Genome-wide programmable transcriptional memory by crispr-based epigenome editing. *Cell*, 184(9):2503–2519, 2021.

[15] Ralf Schmidt, Zachary Steinhart, Madeline Layeghi, Jacob W Freimer, Raymund Bueno, Vinh Q Nguyen, Franziska Blaeschke, Chun Jimmie Ye, and Alexander Marson. CRISPR activation and interference screens decode stimulation responses in primary human T cells. *Science*, 375(6580):eabj4008, 2022.

[16] Max A Horlbeck, Luke A Gilbert, Jacqueline E Villalta, Britt Adamson, Ryan A Pak, Yuwen Chen, Alexander P Fields, Chong Yon Park, Jacob E Corn, Martin Kampmann, and Jonathan S Weissman. Compact and highly active next-generation libraries for CRISPR-mediated gene repression and activation. *eLife*, 5:e19760, sep 2016.

2 Supplementary Figures

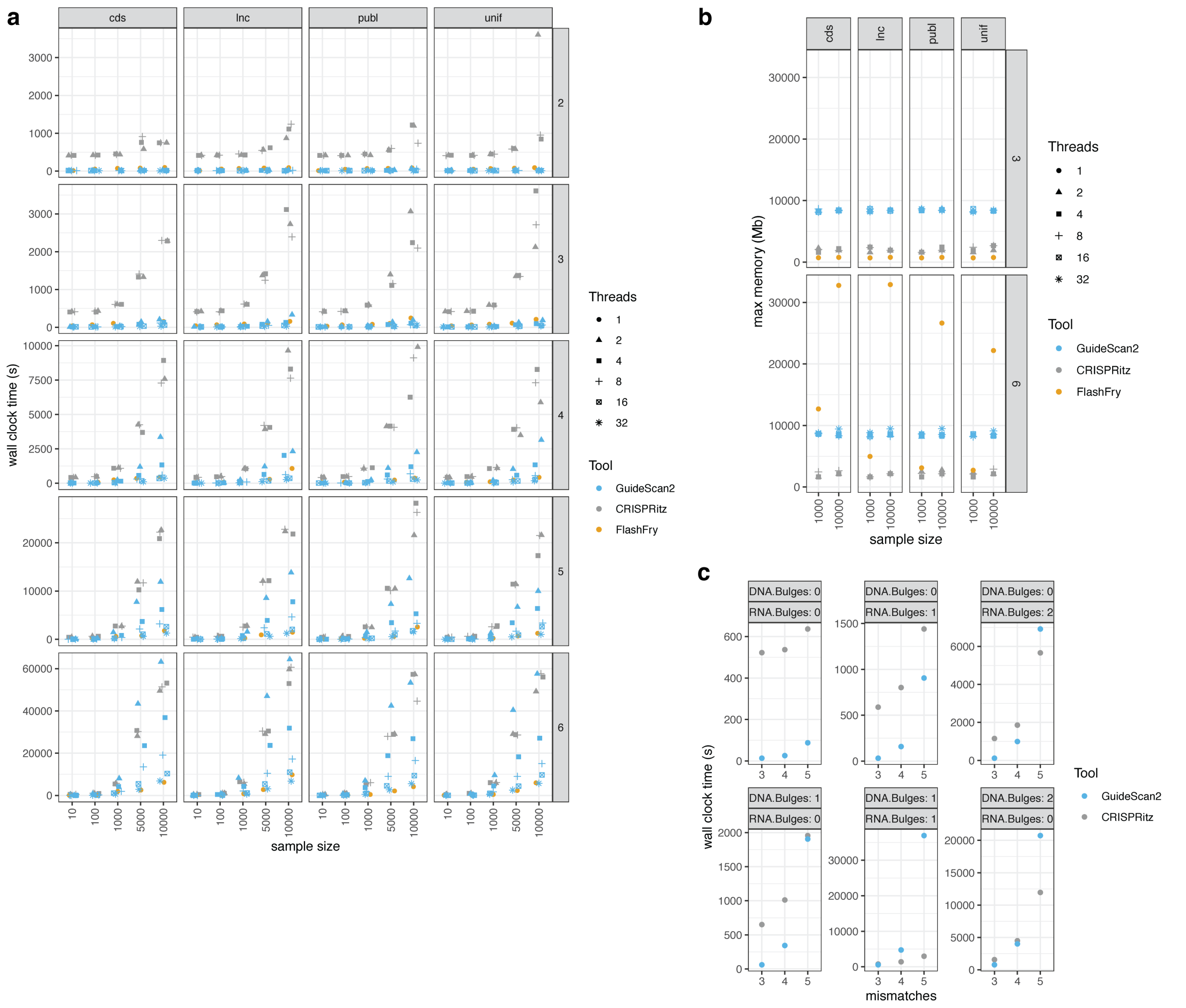

Figure S1: Comparison between GuideScan2 and other methods. (a) Run time comparison between different CRISPR gRNA design command line methods. gRNA off-target search was performed by GuideScan2 and other methods, searching for off-targets with up to a specified number of mismatches (from 2 to 6 mismatches, specified in the plot at the right side of the row). GuideScan2 was run with parallelization with different number of threads. Inputs are random subsets of selected CDS-targeting regions (cds), long non-coding RNA genes (lnc), gRNAs from published libraries (publ), and gRNA sequences chosen uniformly at random (unif). (b) Memory usage comparison between GuideScan2 and other methods. Selected results for the same experiment as in (a). (c) Run time comparison between GuideScan2 and CRISPRitz used for the gRNA off-target search allowing DNA or RNA bulges and variable number of mismatches, for 50 randomly selected gRNAs.

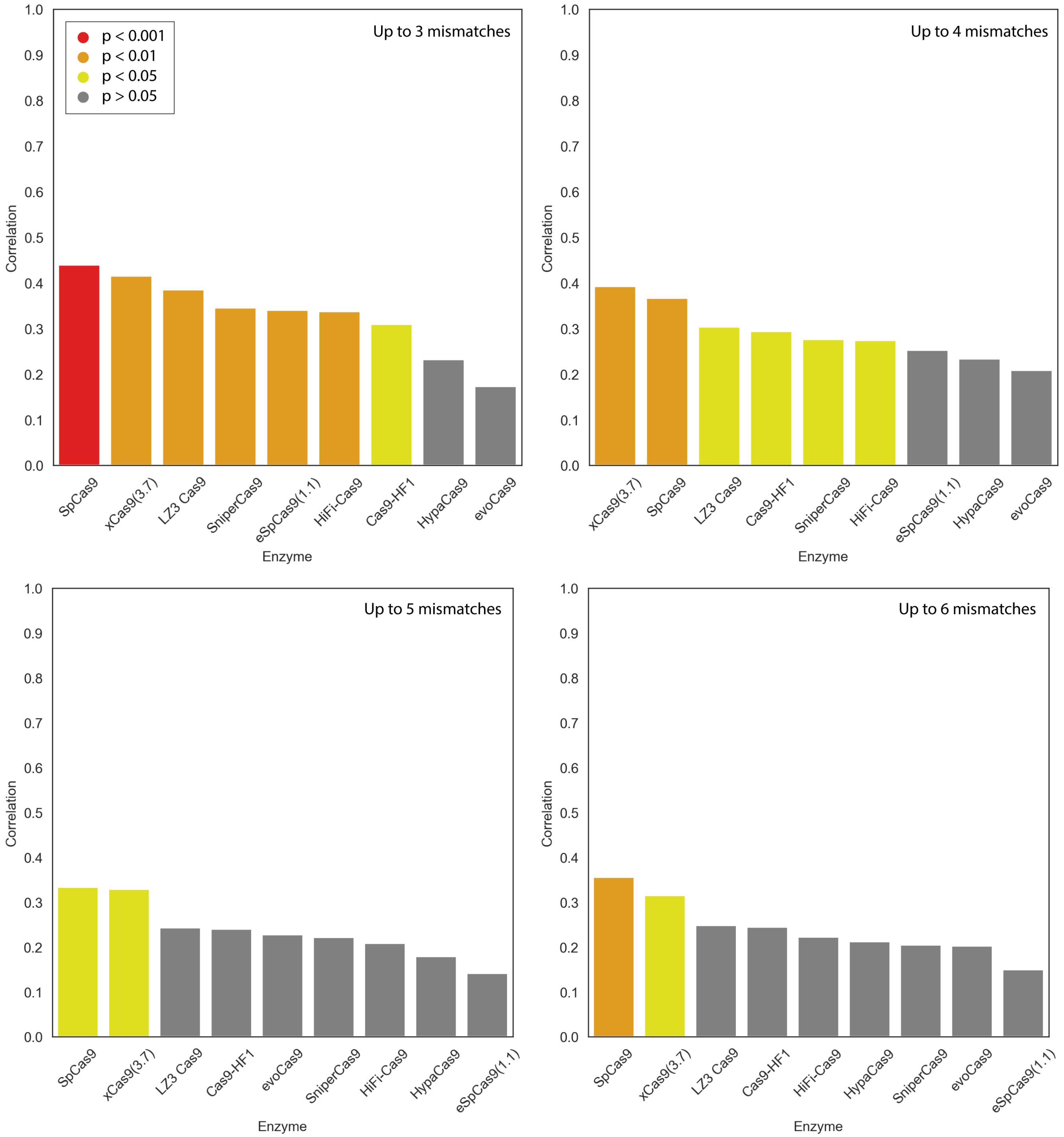

Figure S2: Validation of GuideScan2-estimated gRNA specificities. Correlations of TTISS-derived gRNA specificities with GuideScan2-estimated specificities for 59 gRNAs from the TTISS study [9]. Spearman correlations are calculated for wildtype Cas9 (SpCas9) and for eight Cas9 variants for which TTISS data was generated [9]. GuideScan2 was used to estimate off-targets using the CFD scores [10] and the specificity aggregation formula [11], using all off-targets with up to N mismatches identified by GuideScan2 for $N = 3$ (top left, default for GuideScan2 website), $N = 4$ (top right), $N = 5$ (bottom left) and $N = 6$ (bottom right).

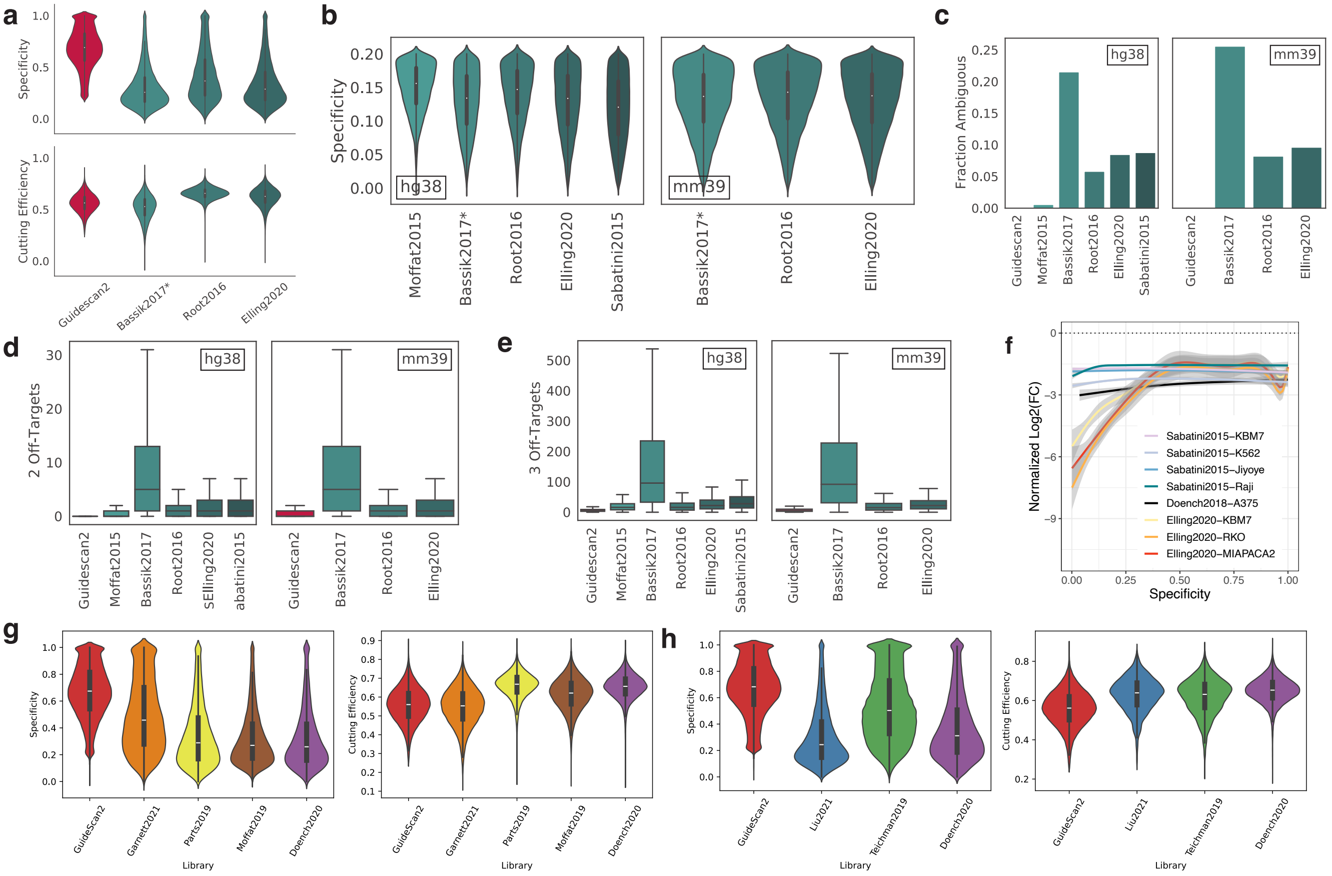

Figure S3: Reanalysis of published CRISPRko libraries. (a) Comparison of new GuideScan2 mouse gRNA library against other libraries by specificity and cutting efficiency (average over gRNAs per gene). (b) Distribution of specificity values (average over gRNAs per gene) at the range between 0 and 0.2 for human and mouse gRNA libraries. By design the GuideScan2 libraries do not have such gRNAs. (c) Fraction of ambiguous genes across libraries. A gene is called “ambiguous” if there is a gRNA intended for that gene that has multiple perfect occurrences (without mismatches) in the genome. By design the GuideScan2 libraries do not have such gRNAs. (d) Boxplots (excluding outliers) of the number of 2-mismatch off-targets per gRNA. (e) Boxplots (excluding outliers) of the number of 3-mismatch off-targets per gRNA. (f) Reanalysis of published essentiality CRISPRko screens for essential genes (**Methods**). Violin plots, boxplots (center line, median; box limits, upper and lower quartiles; whiskers, 1.5 × interquartile range). (g,h) Comparison of specificities and cutting efficiencies (average over gRNAs per gene) between GuideScan2 and an extended list of other gRNA libraries for human (g) and mouse (h).

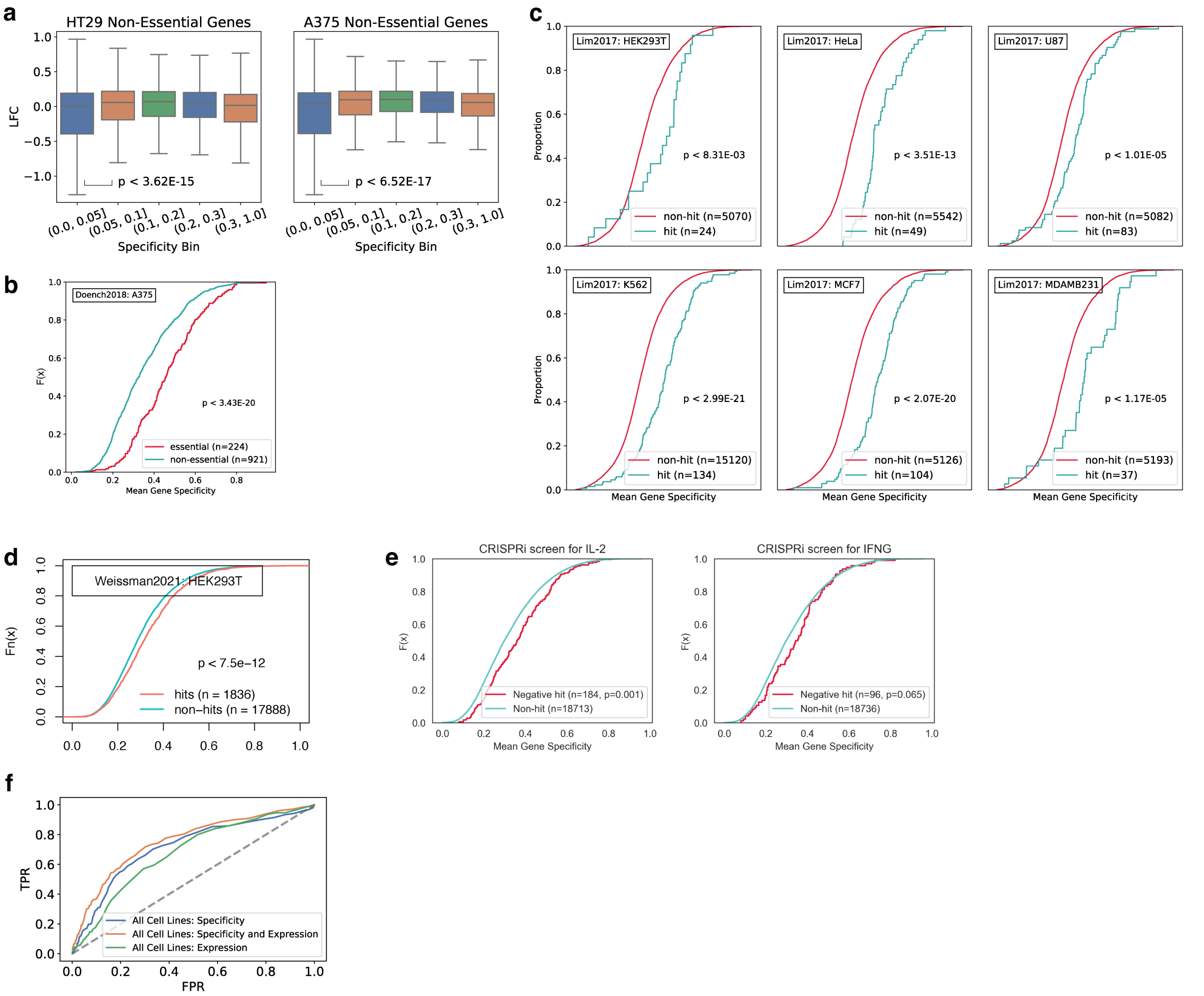

Figure S4: Reanalysis of published CRISPRi screens. (a) Reanalysis of the Doench2018 CRISPRi essentiality screen data. For non-essential genes, the gRNAs with specificity between 0 and 0.05 confer significantly lower LFC values than gRNAs with specificity between 0.05 and 0.1 (Mann–Whitney U test). Boxplots (center line, median; box limits, upper and lower quartiles; whiskers, 1.5 × interquartile range). (b-f) Comparison of gene-level specificity (average over gRNAs per gene) for genes identified by the authors as hits and non-hits in (b) the Doench2018 CRISPRi essentiality screen [12]; (c) the Lim2017 CRISPRi lncRNA screen across multiple cell lines [13]; (d) the Weissman2021 genome-wide CRISPRoff screen [14]; (e) the Marson2022 CRISPRi IL-2 (left) and IFNG (right) screens in T cells [15]. Empirical cumulative distribution function; Kolmogorov–Smirnov test, two-sided. (f) ROC curve for logistic regression classifying between hits and non-hits using gRNA features in the Lim2017 lncRNA CRISPRi screen across all cell lines, following and extending the approach by the authors [13].

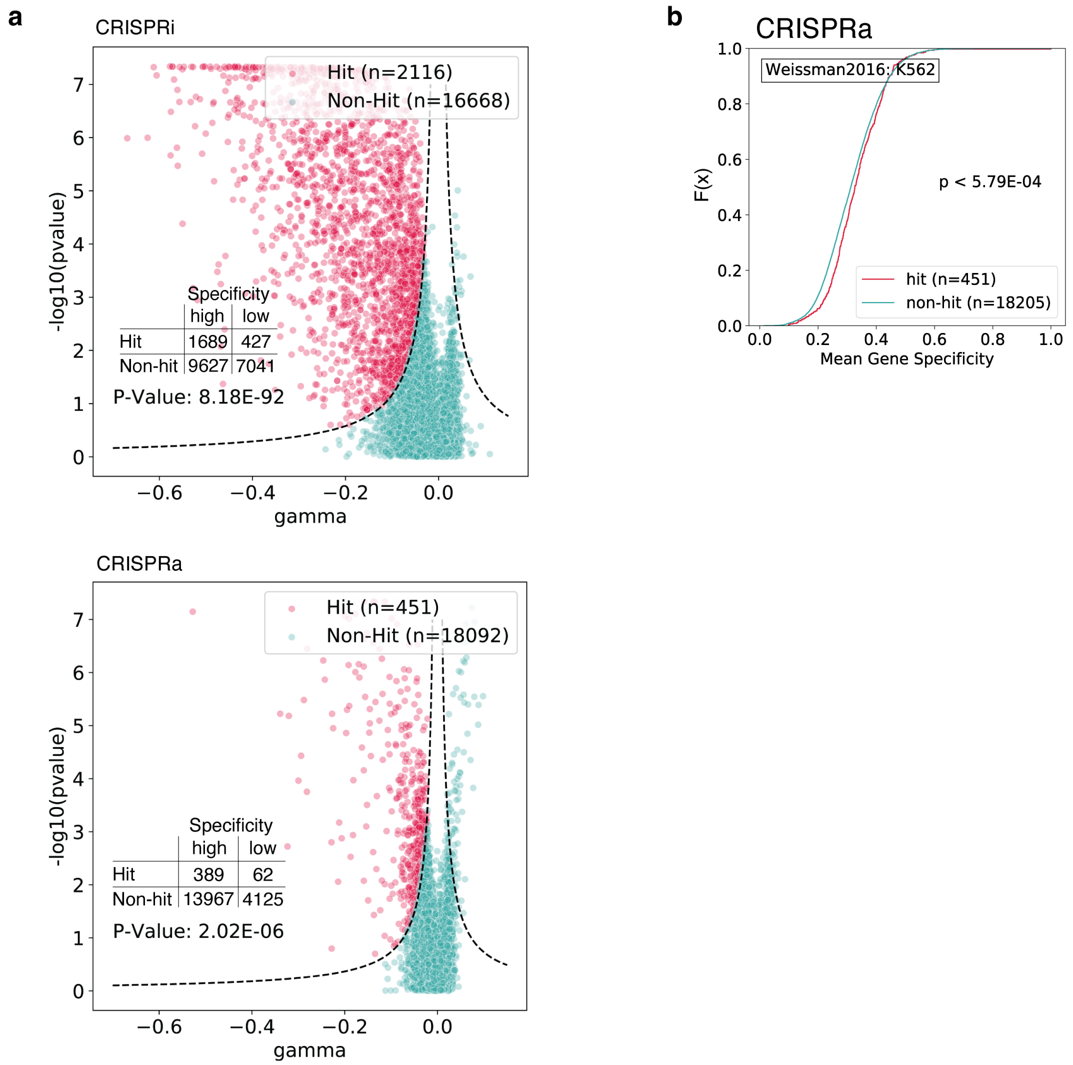

Figure S5: Reanalysis of the Weissman2016 CRISPRi and CRISPRa screens (Horlbeck et al. eLife 2016). (a) Volcano plots for the Weissman2016 CRISPRi and CRISPRa screens [16] showing the definitions of hits and non-hits from the original publication. p-value, Fisher's exact test for enrichment of genes targeted with high-specificity (mean specificity > 0.25) gRNAs among hits. (b) Comparison of gene-level specificity (average over gRNAs per gene) for genes identified by the authors as hits and non-hits in the Weissman2016 CRISPRa screen.

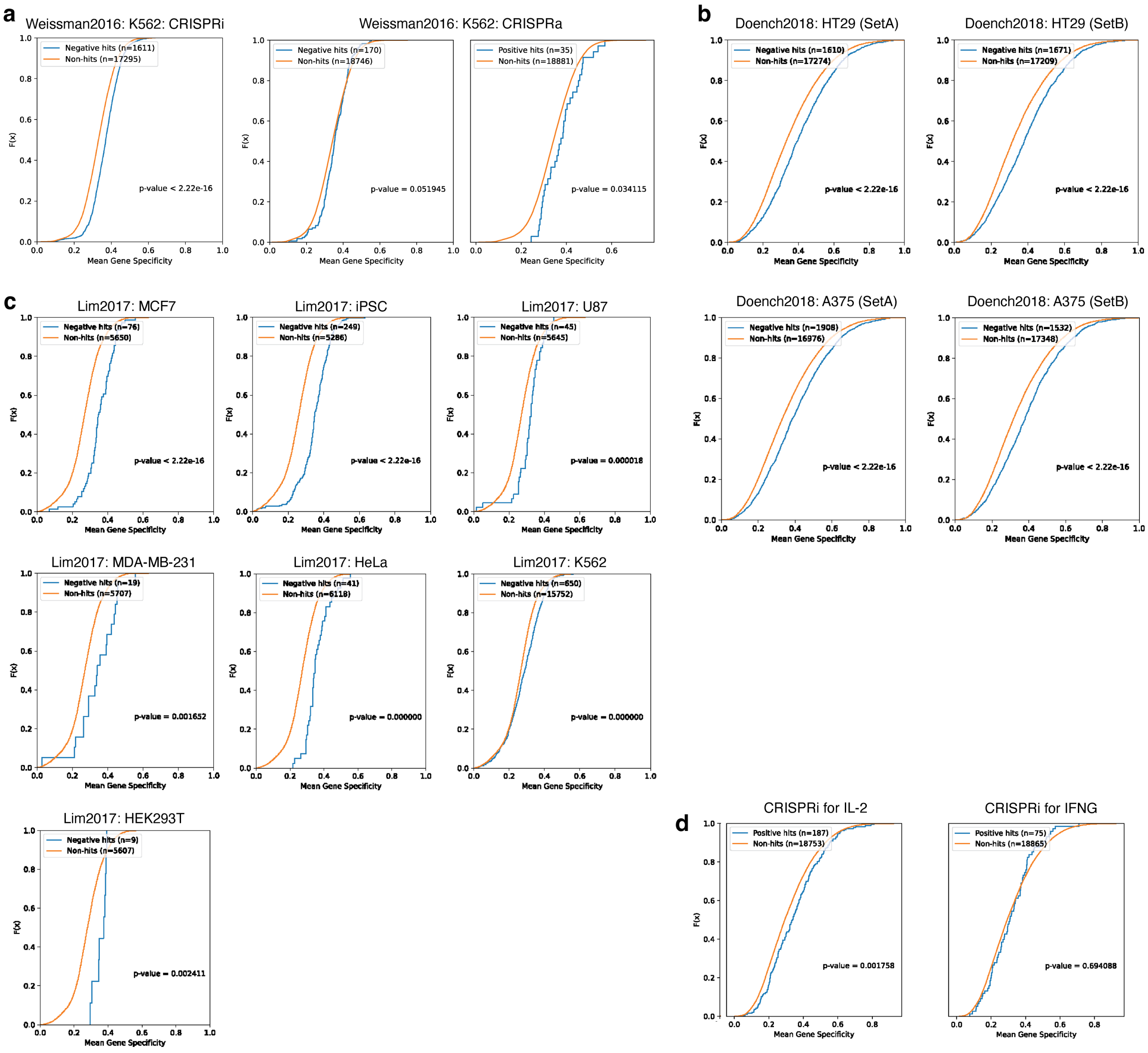

Figure S6: Reanalysis of published CRISPRi and CRISPRa screens with MAGeCK. Comparison of gene-level specificity (average over gRNAs per gene) for hits and non-hits (as identified by MAGeCK) in (a) the Weissman2016 CRISPRi and CRISPRa screens [16]; (b) the Doench2018 CRISPRi essentiality screen [12]; (c) the Lim2017 CRISPRi lncRNA screen across multiple cell lines [13]; (d) the Marson2022 CRISPRi IL-2 and IFNG screens in T cells [15] (note that MAGeCK positive hits for this screen correspond to negative hits as defined by the authors). Empirical cumulative distribution function; Kolmogorov–Smirnov test, two-sided.

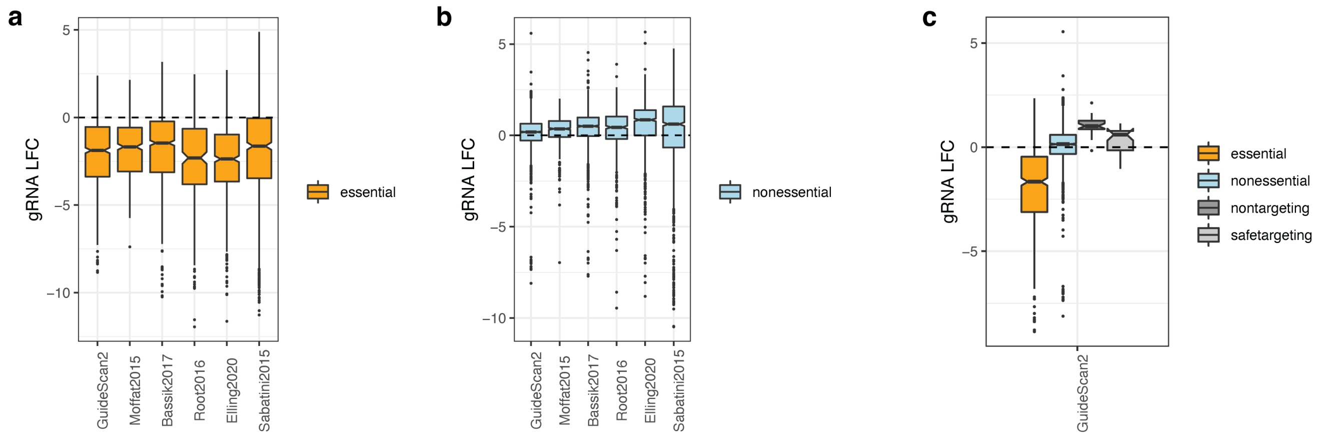

Figure S7: Essentiality screen for validation of the GuideScan2 library. (a, b) Targeting effect for individual gRNAs designed for essential and non-essential genes. (c) GuideScan2 gRNA targeting effect for essential and non-essential genes and for non-targeting and safe-harbor-targeting controls.

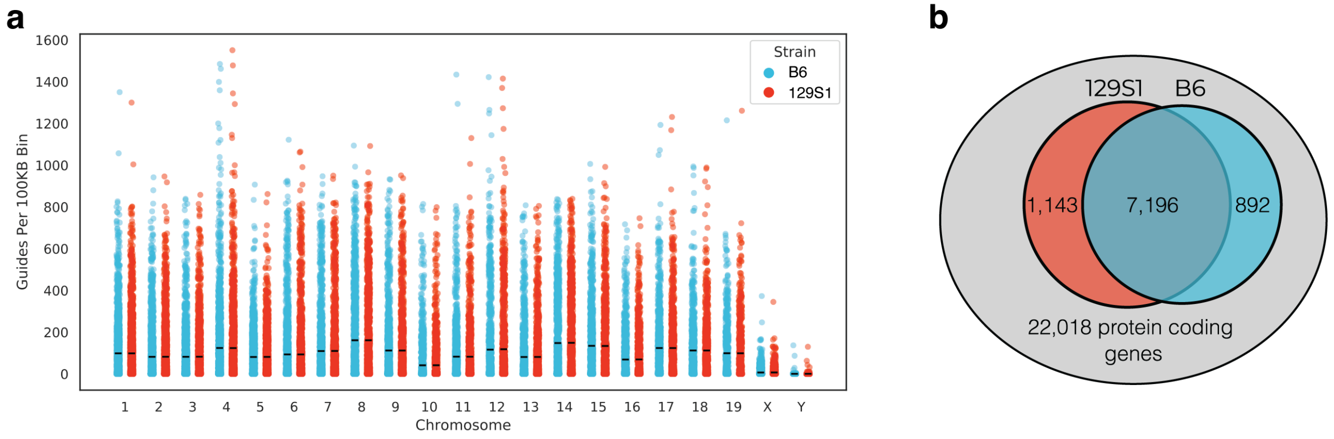

Figure S8: Frequency of allele-specific gRNAs. (a) Genome-wide frequency of C57BL/6- and 129S1/SvlmJ-specific gRNAs (excluding outliers, defined as bins with > 1700 gRNAs, total of 3 bins). (b) The number of protein-coding genes with at least one C57BL/6- or 129S1/SvlmJ-specific gRNA or both.

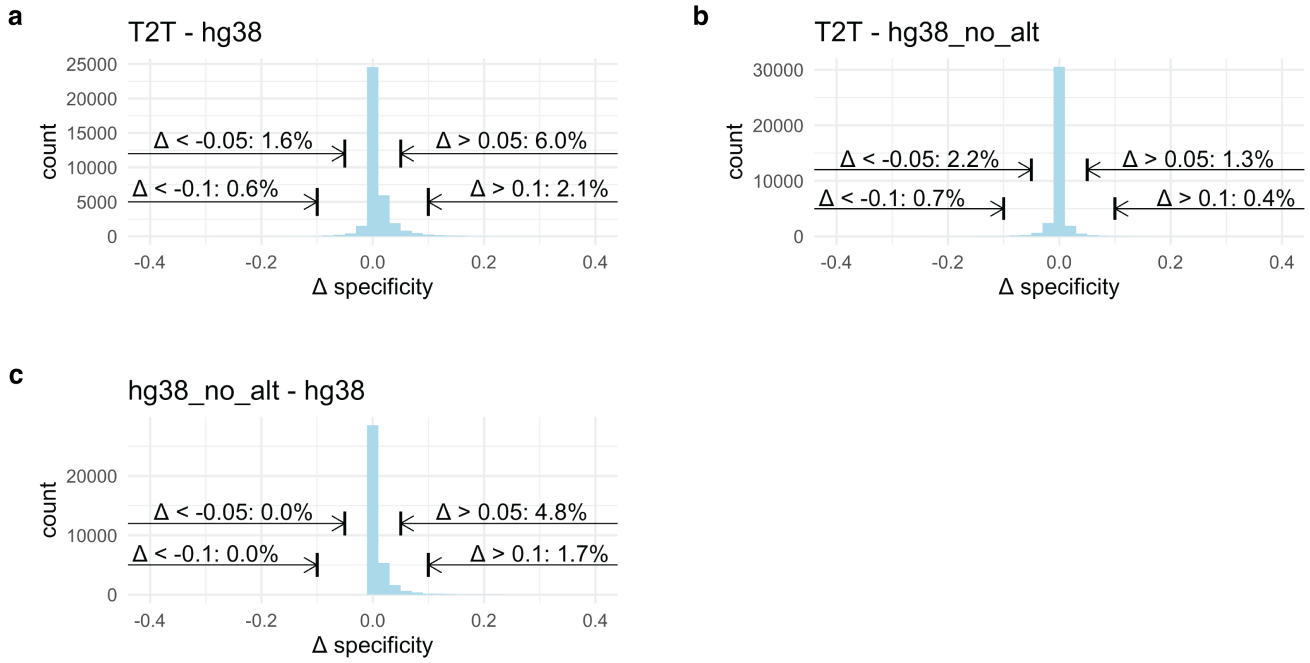

Figure S9: Comparison of CRISPR gRNA specificities with respect to different genome assemblies. Specificities of representative random gRNAs were calculated for different assemblies of the human genome: hg38 (NCBI RefSeq ID GCF_000001405.39 available at `https://www.ncbi.nlm.nih.gov/datasets/genome/GCF_000001405.39/`), hg38_no_alt (GRCh38_no_alt_analysis_set_GCA_000001405.15, hg38 without unassigned scaffolds, the main human genome reference used by ENCODE consortium, available at `https://www.encodeproject.org/files/GRCh38_no_alt_analysis_set_GCA_000001405.15/`), and T2T (GCF_009914755.1, from the Telomere-to-Telomere consortium, available at `https://www.ncbi.nlm.nih.gov/datasets/genome/GCF_009914755.1/`). GuideScan2-constructed genomic indices used for this calculations, as well as corresponding genome-wide gRNA databases, are available at `https://guidescan.com/`. For each gRNA, the difference of specificity values was calculated between (a) T2T and hg38, (b) T2T and hg38_no_alt and (c) hg38_no_alt and hg38. Shown are histograms of distributions of differences across gRNAs, with percentage of differences falling below -0.1, -0.05 or above 0.05, 0.1.
